# Beat-ID: Towards a computationally low-cost single heartbeat biometric identity check system based on electrocardiogram wave morphology

**DOI:** 10.1371/journal.pone.0180942

**Published:** 2017-07-18

**Authors:** Joana S. Paiva, Duarte Dias, João P. S. Cunha

**Affiliations:** 1 Biomedical Research And INnovation (BRAIN), Centre for Biomedical Engineering Research (C-BER), INESC Technology and Science, Porto, Portugal; 2 Physics and Astronomy Department, Faculty of Sciences, University of Porto, Porto, Portugal; 3 Faculty of Engineering, University of Porto, Porto, Portugal; Tianjin University, CHINA

## Abstract

In recent years, safer and more reliable biometric methods have been developed. Apart from the need for enhanced security, the media and entertainment sectors have also been applying biometrics in the emerging market of user-adaptable objects/systems to make these systems more user-friendly. However, the complexity of some state-of-the-art biometric systems (e.g., iris recognition) or their high false rejection rate (e.g., fingerprint recognition) is neither compatible with the simple hardware architecture required by reduced-size devices nor the new trend of implementing *smart objects* within the dynamic market of the Internet of Things (IoT). It was recently shown that an individual can be recognized by extracting features from their electrocardiogram (ECG). However, most current ECG-based biometric algorithms are computationally demanding and/or rely on relatively large (several seconds) ECG samples, which are incompatible with the aforementioned application fields. Here, we present a computationally low-cost method (patent pending), including simple mathematical operations, for identifying a person using only three ECG morphology-based characteristics from a single heartbeat. The algorithm was trained/tested using ECG signals of different duration from the *Physionet* database on more than 60 different training/test datasets. The proposed method achieved maximal averaged accuracy of 97.450% in distinguishing each subject from a ten-subject set and false acceptance and rejection rates (FAR and FRR) of 5.710±1.900% and 3.440±1.980%, respectively, placing Beat-ID in a very competitive position in terms of the FRR/FAR among state-of-the-art methods. Furthermore, the proposed method can identify a person using an average of 1.020 heartbeats. It therefore has FRR/FAR behavior similar to obtaining a fingerprint, yet it is simpler and requires less expensive hardware. This method targets low-computational/energy-cost scenarios, such as tiny wearable devices (e.g., a *smart* object that automatically adapts its configuration to the user). A hardware proof-of-concept implementation is presented as an annex to this paper.

## Introduction

In the past few years, identity recognition methods that are safer and more trustworthy in comparison with the conventional techniques used to date have been extensively explored, even for subject identification among a small group of persons. Identity recognition has found application in several facets of life, including security technology, e-commerce, data protection, entertainment, remote access, voting, health, and social services [1, 2]. However, traditional identity recognition methods, such as passwords or encryption keys, have numerous constraints. These methods can be vulnerable and inefficient for sensing a certain physiological change or simply for identifying a specific person. Consequently, researchers began investigating the possibility of using biometric measures in order to recognize a person. Currently, biometrics-based identity recognition is a rapidly growing research area, not only because of the increasing demands for security in healthcare and law enforcement applications [3], but also for implementation in novel and attractive systems for entertainment applications. In addition to security or other technological industries, the entertainment sector has also been applying biometrics in the industry of user-adaptable objects to make them even more user-friendly [4]. A good example of this link-up is the use of biometrics as a scientific methodology for analyzing and measuring player experience in the video games industry [5]. Indeed, human-interactive technologies have been extensively explored in the past few years to develop *smart objects* and systems capable of interacting with their user (from a restrict group of persons, for example, a family) for a number of different so-called “intelligent” applications within the ever-growing market of the Internet of Things (IoT). These systems are being designed to perform certain actions according to user preferences, emotional and health states, personal needs, etc. [4–9].

Identity authentication using one or more biometric measures ensures identification, authentication, and non-repudiation in information security [2, 3]. Fingerprint, retina, face, iris, and voice recognition were the first technologies to be explored in the field of biometrics [2]. Several recent studies proved that it is possible to identify an individual through morphological features extracted by imaging their ear [10], such as its shape, wrinkles, and ear points. Additionally, as reviewed by Vaidya [2], researchers have also begun to focus on odor, keystroke, or individual gait characteristics to identify persons. Despite these efforts, recent findings revealed that these methods also have several drawbacks, even for person identification among a limited group of subjects. For example, identification of an individual through face-derived features requires the underlying authentication algorithm to analyze specific characteristics such as the width of the nose, the distance between the eyes, and the jaw line. However, these features constantly experience ongoing changes due to modifications in a person’s facial expression, depending on whether the person is reacting to a specific situation, such as smiling or crying, which can introduce highly variable features and compromise the generalization ability of the classifier [2]. Moreover, as the person ages, their face undergoes changes, thereby contributing even more to the large variability in face-derived features [2]. Therefore, results with near perfection accuracy have only been achieved by human facial recognition algorithms (approximately 97%) in highly controlled environments, with the performance of these algorithms highly influenced by several factors such as illumination or subject position [11].

Although it is one of the most mature technologies, fingerprint recognition also has several drawbacks. Its potential can be easily compromised by using a synthetic material, such as gelatin. Additionally, in some situations, such as unconstrained environments, the quality of the acquired data may not be amenable for automated recognition, in which case the majority of input samples are rejected [12]. However, fingerprint recognition algorithms that ensure near perfect performance have already been developed, showing accuracy values of approximately 99% [13].

Performance evaluation is a critical and very particular task in biometric-based algorithms [14]. A challenge related to selecting a biometric algorithm that performs more accurately considering a specific scenario requires the adoption of procedures that are concise with as little bias as possible. In the following subsection, several existing concerns about methods for evaluating the performance of biometric-based algorithms are discussed.

### Performance and quality assessment of biometric-based algorithms

The widely used Accuracy and F-Measure are not the only important metrics for assessing the performance of biometric algorithms. Other parameters, such as the computational requirements (in terms of computational cost), are broadly used for judging identification methods. That is, the computational cost (i.e., the number and type of mathematical operations) is one of the major factors that determines the acceptability of a given biometric system for certain applications [15]. Additionally, statistical measures such as the false rejection rate (FRR) and false acceptance rate (FAR), and the time-derived speed rate (SR) must also be considered [16, 17]. The FRR is defined as the frequency at which it is not possible to match biometric information against any records in a database when a person who is enrolled in the system tries to validate their identity. A biometric system associated with a high FRR can be particularly frustrating, causing unnecessary logs, affecting service, and has a negative impact on productivity [16, 17]. The FAR is a statistical measure that represents the extent to which a person is falsely reported to either match the biometric template or information belonging to another person in the database [16, 17]. Finally, the SR reflects the data processing capability of a method, corresponding to the time for a decision (accept or reject the identification label) being announced by the biometric system (the authentication duration) [17, 18]. According to previous reports, desirable SR values range between 6 and 10 seconds. However, only very recently have a few biometric systems capable of meeting these speed standards been developed [19–21]. Table 1 provides the current range values for the SR of the most commonly used state-of-the-art biometric methods. In fact, the response speed of these types of techniques remains a challenging topic in the field of biometrics.

**Table 1:** Speed Rate (SR) range values for some state-of-the-art biometric techniques.

Biometric Method Type	Speed Rate (SR)	Ref. Nr.
Facial Recognition	3-30 seconds	[19, 22]
Speech Recognition	5-30 seconds	[2, 3, 19, 23]
Vein Pattern Recognition	2-40 seconds	[19, 24, 25]
Hand Geometry Recognition	≈0.6 seconds-10 minutes	[26–28]
Iris Recognition	2-5 seconds	[21, 29, 30]
Signature Recognition	3-5 seconds	[20]
Fingerprint Recognition	20 seconds-1 minute and 45 seconds	[16, 31, 32]
Face + Fingerprint Recognition	≈5 minutes	[33]
Retina Recognition	6-15 seconds	[23, 34]

The generation of training and test sets to evaluate the performance of biometric-based algorithms does not follow the regular principles of a *machine-learning* problem, in a way that biometrics training procedures always require to be composed of samples of each subject enrolled in the biometric system [15, 35–44]. The majority of classifier performance evaluation schemes for differentiating classes across subjects (e.g., to identify a pathological ECG signal from non-pathological signals independently from the subject under observation) require the proposed algorithm to be tested on different subjects than those used for training (by applying the *leave-one-out cross validation* [45] instead of the *k-folds cross-validation* method, for example). In contrast, the biometrics algorithm needs prior knowledge of the information of all subjects to be identified, in order to be known by its universe of choices. This latter requirement is common across several biometric systems that used *machine-learning* concepts for solving identity recognition/validation problems during the past 15 years (see Table 2) [14, 15, 35–44, 46]. This problem relates to the particular attribute of biometric-based classification problems: each one of the subjects considered to be identified is one of the classes in the *pattern recognition* problem. Since each “subject” represents a “class” of the problem, if no information about a subject is provided to the classifier, it would be as though such a “class” does not exist for the classifier; therefore, the latter would be unable to detect it. Extrapolating for the case of a simple binary differentiation between “pathological” and “non-pathological” samples, not providing data from one subject to be identified in the training stage of a biometric-based algorithm, is such that expecting that an algorithm is able to detect “non-pathological” signals, after only having access to the “pathological” class information. When the rules behind *machine-learning* problem-solving strategies are closely followed, the differences in the physiological properties of humans, as the basis for identity recognition by biometric devices, can bias the test results between test user groups. Therefore, Holmes et al. [47] presented a balanced solution to evaluate biometrics classifier’s accuracy, adopting a performance evaluation strategy with as little bias as possible. They proposed an evaluation scheme compatible with “simulating” a scenario of numerous attempts by each user, for example by collecting performance results for several runs, using different sets for training and testing between each run [47]. This approach is adopted in the present study.

**Table 2:** List of examples of biometric algorithms tested after all subjects to be identified were enrolled in the system, across the last 15 years.

Year and Ref.	Authors	Biometric Data Type
2001, [39]	L. Biel, O. Pettersson, L. Philipson et al.	ECG
2002, [48]	S. Prabhakar and A. Jain	Fingerprint
2003, [49]	M. Munich and P. Perona	Written Signature
2005, [40]	S. Israel, J. Irvine, A. Cheng et al.	ECG
2005, [50]	D. Woodard and P. Flynn	3D Finger Surface
2006, [22]	C. Chen and C. Chu	Face and Iris
2006, [51]	H. Çetingul, E. Erzin, Y. Yemez et al.	Speech
2006, [44]	R. Palaniappan and K. Ravi	EEG*
2007, [52]	I. Kakadiaris, G. Passalis, G. Toderici et al.	Face Recognition
2007, [43]	K. Phua, J. Chen, T. Dat et al.	Heart Sound
2007, [53]	L. Wang, G. Leedham and S. Cho	Hand Vein Patterns
2007, [54]	G. Wübbeler, M. Stavridis, D. Kreiseler et al.	ECG
2008, [37]	Y. Singh and P. Gupta	ECG
2008, [55]	S. Ziauddin and M. Dailey	Iris
2009, [42]	F. Agrafioti, D. Hatzinakos	ECG
2009, [41]	J. Irvine, S. Israel	ECG
2010, [56]	M. Derawi, C. Nickel, P. Bours et al.	Gait
2011, [36]	A. Lourenço, H. Silva and A. Fred	ECG
2012, [38]	C. Ye, B. Kumar, M. Coimbra	ECG
2013, [35]	M. Yang, B. Liu, M. Zhao et al.	ECG

*EEG—Electroencephalogram.

This particular mode of generating training data for biometric pattern recognition problems—including data provided by all the subjects from the selected universe—has not yet, to the best of our knowledge, been a defined designation in the literature. In fact, it was merely vaguely referred to by, for example, Jain et al. [57], who grouped the process designation of comparing data acquired online with information stored for an enrolled person for *verification* or *identification*, by the term **“recognition issue”**. Let this procedure be termed **“Subject-Match Enrollment”**, since training the classifier with the biological “signature” of each subject mimics subject template enrollment in the biometric system.

**Fig 1:**
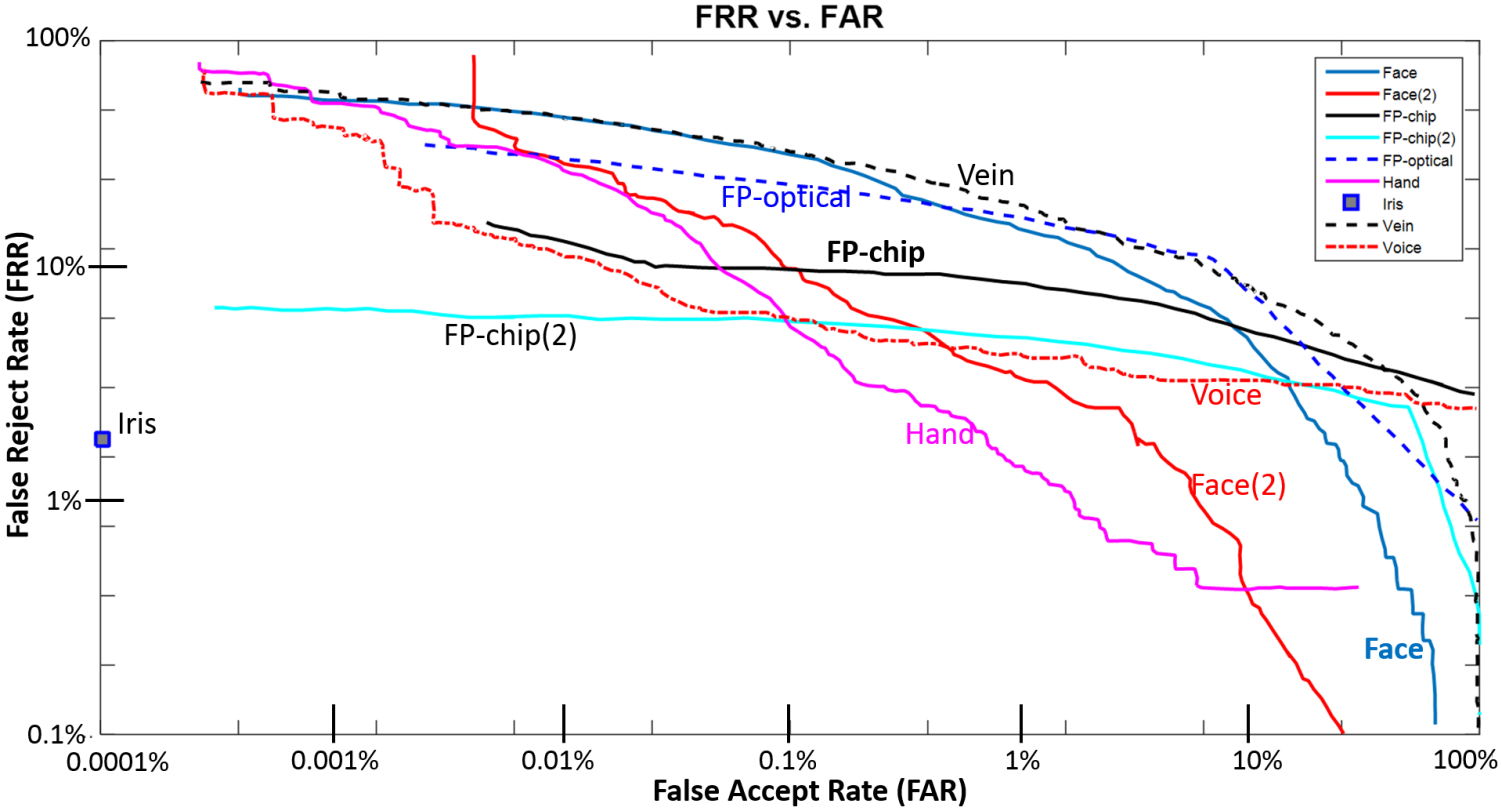
Schematic representation of FRR versus FAR curve for several state-of-the-art biometric techniques. Face and Face(2)—two face-recognition systems validated using different devices; FP-chip and FP-chip(2)—fingerprint recognition through a chip sensor tested with two different methods/devices; FP-optical—optical-based fingerprint recognition; Hand—hand-based biometrics; Iris—iris-based recognition; Vein—vein patterns-based recognition; Voice—voice recognition. The devices/systems used to validate each one of those biometric techniques are referred to and described in the study of Mansfield et al. [17]. Graphic generated based on the results of Mansfield et al. [17].

In Fig 1 we present a schematic representation of FRR versus FAR curve values regarding several state-of-the-art biometric techniques, based on the study of Patrick et al. [16, 17]. The relationship between FRR and FAR (Fig 1) regarding the fingerprint recognition method reveals that this technique performs best for low acceptance rates, in comparison with the remaining methods. However, fingerprint recognition has a relevant drawback that significantly affects the performance of the method: its high FRR. Independent of the detection performance, the system rejects about 10% of its input values [16, 17]. Voice, hand, and face recognition behave highly variably in comparison with fingerprint recognition. These three techniques tend to reject almost all of the input samples in order to ensure performance values similar to fingerprint recognition. The iris system proves to be the best relative to all the techniques, with only 1.8% of false rejections. However, the complexity of this method increases significantly in comparison with the remaining methods.

Researchers have begun to investigate novel biometric technologies for identifying individuals, besides improving the accuracy associated with existing biometric identification systems. Recently, some researchers proposed identity recognition methods based on individual touching behavior, by exploiting biometric features such as position, pressure, or size, when a subject simply inputs a pattern password into a touch screen [58, 59]. However, this novel method also has implications that compromise its performance. The fact that it is only focused on the collection of pattern data provided from a single type of sensor and considering that an individual can hold/touch a certain object in a different manner on different occasions, compromises its feasibility. Therefore, scientists endeavored to develop a system capable of recognizing its user through information provided by a more robust biometric signature that could not be affected by sudden and significant changes observed in their emotional state, age, acquisition location, subject position relatively to sensor, etc.

### ECG-based biometrics

**Fig 2:**
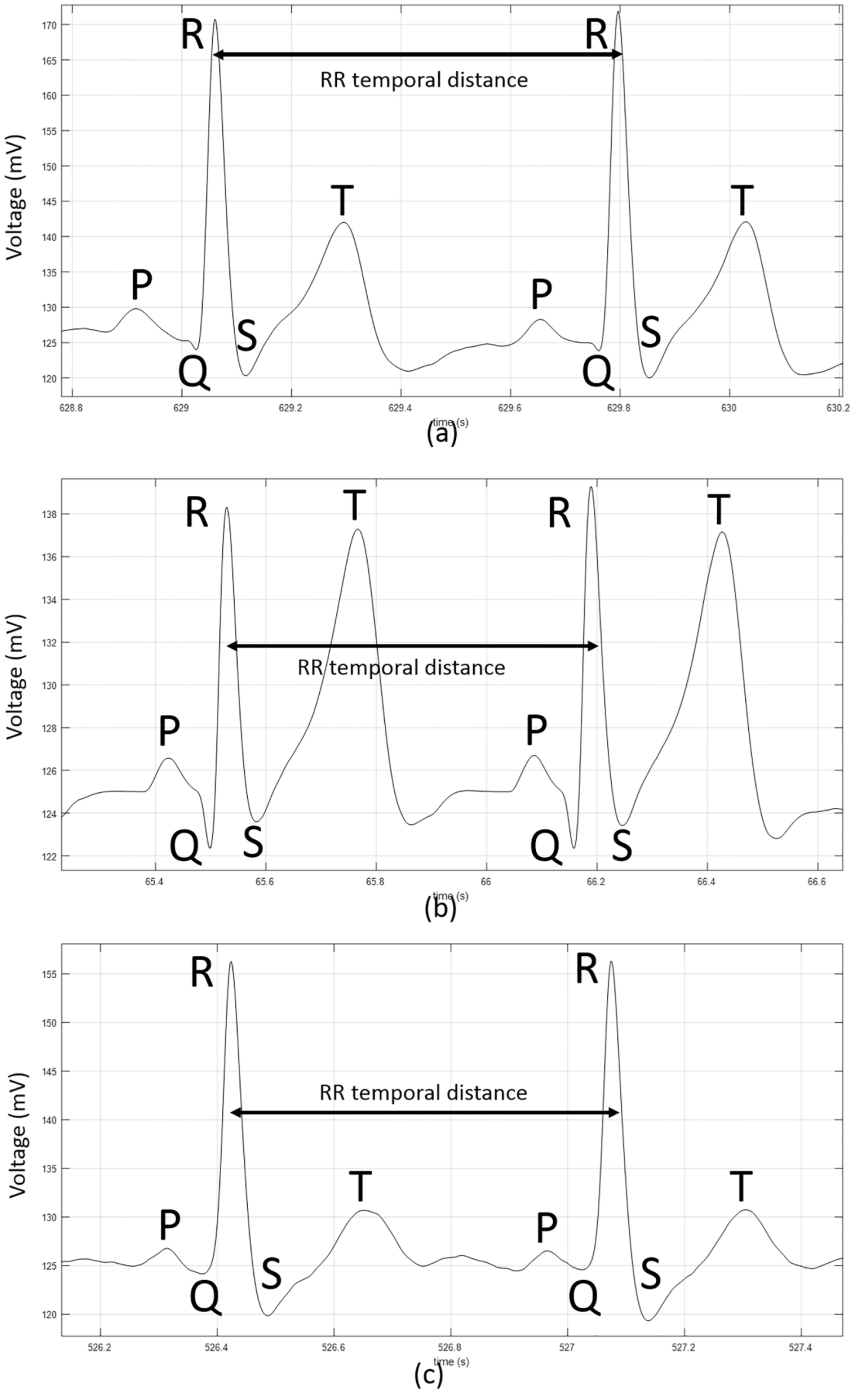
Three examples of ECG acquisitions from three subjects (different from the ones whose ECG information was used in the classification) acquired using the one-lead VitalJacket^™^ wearable platform [72, 73], with fiducial points P, Q, R, S, and T that define the heartbeat morphology. (a) Male subject; 59 years; *RR* = 736 ms. (b) Male subject; 25 years; *RR* = 660 ms. (c) Female subject; 28 years; *RR* = 656 ms.

For the aforementioned reasons, the application of electrocardiography (ECG) for biometric purposes has been studied in the past few years [3, 39, 60–63], due to the recent advances in ECG processing techniques. In fact, the development of complex network theories explaining the time-varying phenomena of ECG (e.g. RR temporal interval variability along time) and other biological data has been a significant contribution for the understanding and improvement of ECG and other biosignals’ information extraction techniques [45, 64–66]. It has been established that ECG, besides providing liveliness detection, is strongly correlated to the subject’s arousal level [3, 36, 63]. Additionally, an ECG signal is difficult to steal and impossible to mimic, because it is unique and intrinsic to each subject. ECG describes the electrical activity of the heart, providing information about the heart rate, rhythm, and morphology [15, 67]. ECG is recorded using electrodes attached to the body surface [3]. Its waveform reflects the sequential depolarization and repolarization of the right and left atria/ventricles. A typical ECG wave provided by a healthy subject is composed of the following fiducial points: P, Q, R, S, and T (a P wave, a QRS complex, and a T wave)—see Fig 2. The P wave usually has a positive polarity and is related with the atrial depolarization. Particularly, the temporal interval between the beginning of P wave and R point transduces the duration that an electrical pulse takes to travel from the sinoatrial node (associated with the heart pacemaker rhythm) to the ventricle [68]. The QRS complex corresponds to the ventricular depolarization (both the left and right ventricles), which has the largest amplitude of the ECG waveform. It is representative of how long the ventricles take to depolarize [68]. It is intrinsically dependent on the heart rate, since the lower the heart rate, the wider the QRS complex, due to the influence of the heart rate in the signal conduction speed through the ventricles (which in that case decreases) [68]. QRS complex is usually asymmetric when comparing QR and RS temporal distances. However, this asymmetry is not constant and also varies based upon changes in heart rate and respiration, for example [15, 67, 68]. The T wave is also highly dependent on the heart rate, because it reflects the ventricular repolarization. It can extend for about 300 milliseconds after the QRS complex [15, 39, 40, 67, 69]. Normal values for interbeat duration (temporal distance between consecutive R points—please see Fig 2)—are between 300 milliseconds and 2 seconds [68].

Physiological and geometrical differences, such as differences in the heart position, size, conductivity of various cardiac muscles, cardiac activation order, and physical conditions are the main physiological factors contributing to the heterogeneity of ECG morphology among individuals [70, 71]. For example, athletes or individuals that usually practice sport could have an increased ST temporal distance [68]. The QT interval, which is defined between the onset of the QRS complex and the end of the T wave and that transduces the time duration between the start and the end of a ventricular depolarization, also varies upon age and gender or drugs consumption habits [68]. Other aspects that are not directly related with the temporal distances between fiducial points, such for example fiducials’ height or trace form also change upon certain factors. In fact, as the heart rate increases (e.g., when a subject is stressed or anxious) the T wave also increases in height and becomes more symmetrical. Additionally, in certain groups of persons (e.g., athletes), the T wave is often inverted in their ECG [68, 70, 71]. In Fig 2 three examples of ECG acquisitions from three different subjects at 500 Hz and from one-lead using the VitalJacket^™^ wearable platform [72] are provided, illustrating ECG morphology differences among different subjects. However, morphology-derived ECG characteristics involving more complex information such as heart wave inclination angles or ECG wave peaks amplitude values are noisier than temporal distances and can introduce errors into the classifier’s decision function for biometric purposes. In fact, inclination angles, width and/or amplitude of ECG waves are highly dependent on the acquisition sensor characteristics (e.g., amplitude gain) or skin conductance.

#### Types of ECG-based biometric algorithms

Scientists classified ECG-based biometric techniques into two types: those that are based on the detection of fiducial points, and those based on the extraction of features in the frequency domain. The first type, which supposes direct time-domain feature extraction, is the first ECG-based biometric method reported in the literature [15]. The features used in this type of classification are only based on the morphology of the ECG, because of its simple extraction. These features are mostly based on the location of the ECG fiducial points. Some examples are the P wave duration, P amplitude, QRS duration, QRS amplitude, T duration, T amplitude, temporal intervals, and amplitude difference between fiducial points. Currently, most of these time-domain features are used for cardiovascular diagnosis [15]. As referred above and according to several studies, some of the characteristics of the ECG waveform could be permanent, distinctive from person to person or between different groups of persons, and sufficiently stable to correctly identify a subject, thereby enabling its usage in biometrics [67, 74].

One of the problems of ECG-based biometric systems that use time-domain features is the time-varying nature of ECG waves. Indeed, the morphology of an ECG signal acquired even for a few seconds can, from time to time, even vary for the same subject [15], due to sudden changes in the cardiac heart rate, which is controlled by the autonomic nervous system (ANS) [40]. The ANS is composed of sympathetic and parasympathetic systems. The former system is responsible for the stimulation of the cardiac system by increasing the rate of the sinoatrial node, increasing the conductivity of cardiac cells, and increasing the force of contraction, whereas the latter system has the opposite effect. Activation of the sympathetic system results in the reduction of the interbeat interval (temporal distance between consecutive R points) variability, and a width reduction in the P and T complexes due to an increase in conductivity [40]. Indeed, the time intervals among the fiducial points change with varying heart rate [40, 75]. However, the differences in the ECG morphology induced by different physiological conditions (e.g., exhaustion, stress, relaxation, and anxiety), would not compromise the performance accuracy of ECG-based biometric systems, if some kind of normalization regarding the heart rate was to be applied to the temporal distances between fiducial points [35, 36, 40, 69, 70, 75, 76]. Despite of all temporal distances between fiducials be affected by heart rate, the time intervals *ST* and *QT* are even more influenced by heart rate [77, 78]. In fact, there are evidences that *QT* distance is highly influenced by heart rate, since it reflects the interval between the onset of electrical activation and its recovery, transducing the balance between the sympathetic and parasympathetic nervous systems [79]. There are several types of normalization methods in literature to remove the influence of heart rate on the temporal distances between fiducial points [68, 79–82]. The method most commonly used for fiducials’ distance heart rate normalization is the *Bazett Formula* [80, 81]. However, this formula is only suitable for heart rate values within the range of 60-90 beat per minute (i.e. RR temporal distances between 666ms and 1000 ms) [82] and should not be used for correcting fiducials’ temporal distances when subject’s heart rate/RR distance is not within these limits. All the other normalization techniques are, in general, defined considering that the average heart rate dependency of fiducials’ intervals could be removed by multiplying each temporal interval by *α* = (*RR*)^*x*^, in which *x* is a given factor that must be optimized for each particular subject and *RR* corresponds to the subject-specific *RR* interval and not to the group average value (mean across subjects). As it was expected, in the majority of the cases, this *α* normalization value works over a limited range, being subject-dependent to some degree, over and above confounding and highly variable factors such as age, gender or physical condition [68, 81]. Additionally, the majority of the studies in which information about the temporal distance between fiducials points are analyzed, did not correct all the temporal features for the influence of the heart rate. Usually, only the QT temporal interval is corrected for heart rate [68, 79–82].

The majority of ECG-based identity recognition algorithms developed to date are of the frequency-related type. Therefore, computationally they are highly demanding and time consuming [71, 83]. Several researchers have used both types of features for identifying individuals [69, 71]. However, the latter types of algorithms are even more computationally demanding in comparison with those that use only one type of feature.

In a world in which small-scale technology has become extremely valued, where IoT mobile and wearable technologies are emerging as the next important markets, most of all with applications in the entertainment and gaming industries, simpler biometric algorithms are required to overcome processing and storage limitations. Recently, several small-scale interactive systems designed to be controlled by biometric inputs were developed (e.g., user-adaptable toys [84], biofeedback systems [9], and video games based on brain-computer interfaces (BCI) [85]—see Tables 3 and 4 for a review of recently developed systems that use biometric technologies for entertainment purposes).

**Table 3:** Review of biometric-related technologies used in entertainment applications and for other purposes—Part I.

Year	Reference Nr.	Title	Authors	Subject	Brief Description
2006	[86]	*Method and apparatus for electrobiometric identity recognition*	D. Lange	ECG Biometrics	A method and apparatus for identifying a person through ECG signal, by computing the correlation coefficient between the acquired signal and a template of the specific subject.
2007	[87]	*Remote health-care monitoring using Personal Care Connect*	M. Blount, V. Batra, A. Capella et al.	ECG Biometrics	Real-time health monitoring system which, besides monitoring the patient heart, also includes an authentication process using the ECG signal.
2011	[88]	*Using a shape-changing display as an adaptive lens for selectively magnifying information displayed onscreen*	S. Fyke and N. Ladouceur	Adaptable Object with Biometrics Input	A touch-sensitive display containing an array of shape-changing zones, which can be independently actuated to form a magnifying lens over an onscreen object of interest (e.g., the lens can be used to magnify a route displayed on a map, or simply to zoom in on a point of interest).
2011	[7]	*Base frame for game using an electric probe in adaptable configurations*	L. Lenkarski and J. DeCarolis	Adaptable Toys (No Biometrics)	A user-adaptable universal base structure that can be used for manufacturing different versions of an electromechanical game.
2011	[85]	*Unveiling the biometric potential of Finger-Based ECG signals*	A. Lourenço, H. Silva and A. Fred	ECG Biometrics	A finger-based ECG biometric system.
2011	[89]	*Clinical data privacy and customization via biometrics based on ECG signals*	H. Silva, A. Lourenço, A. Fred et al.	ECG Biometrics	A framework for continuous identity verification for Healthcare Information Systems (HIS).
2011	[90]	*Biometric Interface for a handheld device*	G. Weising	Biometrics Input	A method and system for processing electrocardiographic data (ECG signals) applied as input to an interactive program. The settings or simply the state of the interactive program can be modified based on the biometric information.
2012	[8]	*System and methodology providing adaptive interface in an industrial controller environment*	J. Baier, D. Wylie, D. Vasko et al.	Adaptable GUI	A system and methodology providing an adaptive user-friendly GUI adequate for an industrial control environment. This user interface can operate across several software and/or hardware platforms.

**Table 4:** Review of biometric-related technologies used in entertainment applications and other purposes—Part II.

Year	Reference Nr.	Title	Authors	Subject	Brief Description
2012	[6]	*Shape-adaptable surface for an audio port*	S. Fyke, N. Ladouceu, J. Griffin	Adaptable Audio Port	An apparatus for providing a shape-adaptable surface for an adaptable audio port. The system includes an audio port, a shape-adaptable surface with several portions, a plurality of sensors coupled to the surface, and a processor operatively coupled to the shape-adaptable surface and the plurality of sensors.
2013	[91]	*Finger ECG signal for user authentication: Usability and performance*	H. Da Silva, A. Fred, A. Lourenço et al.	ECG Biometrics	A finger-based ECG biometric system that uses the signal collected at the fingers. The system comprises a minimally intrusive 1-lead ECG setup recurring to Ag/AgCl electrodes without gel as interface with the skin.
2013	[92]	*Device and method for continuous biometric recognition based on electrocardiographic signals*	H. Silva, A. Lourenço, A. Fred	ECG Biometrics	A finger-based ECG biometric device allowing identity recognition in an uninterrupted way—technology applicable to clinical data protection, vehicles, tablet computers, etc.
2014	[93]	*System and method for enabling continuous or instantaneous identity recognition based on physiological biometric signals*	F. Agrafioti, F. Bui and D. Hatzinakos	ECG Biometrics (Safe and Security)	Biometric security system and method able to authenticate one or more individuals using physiological signals, such as ECG, electroencephalogram (EEG), photoplethysmogram (PPG), and blood volume pressure (BVP).
2014	[9]	*Biometric sensing device with adaptive data threshold, a performance goal, and a goal celebration display*	C. Brumback, D. Knight, J. Messenger et al.	Biofeedback	Monitoring device that receives one or more biometric inputs and tracks the completion progress towards one or more biometric performance goals.
2014	[94]	*Communication apparatus using biometrics*	J. Hjelm and J. Soderberg	ECG Biometrics	An apparatus allowing connection to a network after authentication of its user. This system comprises a subscription module stored in memory and a controller that obtains biometric information of the user by using a sensor and compares this information to the identification information in the subscription module.
2014	[95]	*Multi electro-biometric user recognition*	D. Lange	ECG Biometrics	A processor-based device that contains a pair of contacts that can be used to collect two different types of human biometric data. This data can therefore be processed and used to authenticate the user device.
2014	[96]	*Electro-biometric identification*			

#### State-of-the-art of short-term ECG biometric methods

Efforts are being directed toward developing an ECG based-algorithm for biometric purposes capable of recognizing a person in as brief a time as possible [1].

Regarding the state-of-the-art on short-term ECG-based biometric systems that are currently available, recently, Wübbeler et al. [54] proposed an algorithm that ensured a classification accuracy of 97% by using the information extracted from 10 heartbeats. Can Ye et al. [97] also developed a method that ensures a good accuracy for subject identification using ECG acquisitions of only 6 seconds (approximately six to eight heartbeats). However, it applies complex feature extraction methods and uses 26 features for training the classifier, implying a high computational cost. Additionally, Kang et al. [98] proposed, very recently, an ECG biometric algorithm that uses five heartbeats for identifying a subject, despite their implementation being complex including complex mathematical operations and a computational cost incompatible with simple low-cost hardware microcontrollers. To the best of our knowledge, no other researchers have proposed a method capable of identifying a person using a smaller number of heartbeats, with a computational complexity degree compatible with it being embedded in a simple hardware module.

In this paper, we propose a method we named ***Beat-ID*** for identifying a person, by using only the morphological features of their heartbeat based on three distance measures among ECG fiducial points in the time domain. By capturing an ECG wave and extracting the temporal distances between the Q, R, S, and T fiducial points and using machine-learning techniques, this algorithm is capable of automatically identifying a person using only the information provided by one or two heartbeats. It was designed to be mainly used for a restricted group of persons (e.g., a family) for entertainment, gaming purposes and/or bio-feedback systems (e.g., to be embedded on a smart object, which after its user be detected, could be able to interact with the user in an intelligent way, for example, by changing its apparatus in terms of shape, color, drawings, etc). Algorithm’s requirements were determined to allow its incorporation in a simple microcontroller. Naturally, this simplicity degree could not be comparable to the complexity of high cost methods such as iris and retina recognition. However, the purpose of the *Beat-ID* is to ensure a simple and as fast as possible ECG recognition system for a restrict group of persons. It was designed to meet the single heartbeat performance, for the cost of a more limited subject’s sample size.

## Materials and methods

**Fig 3:**
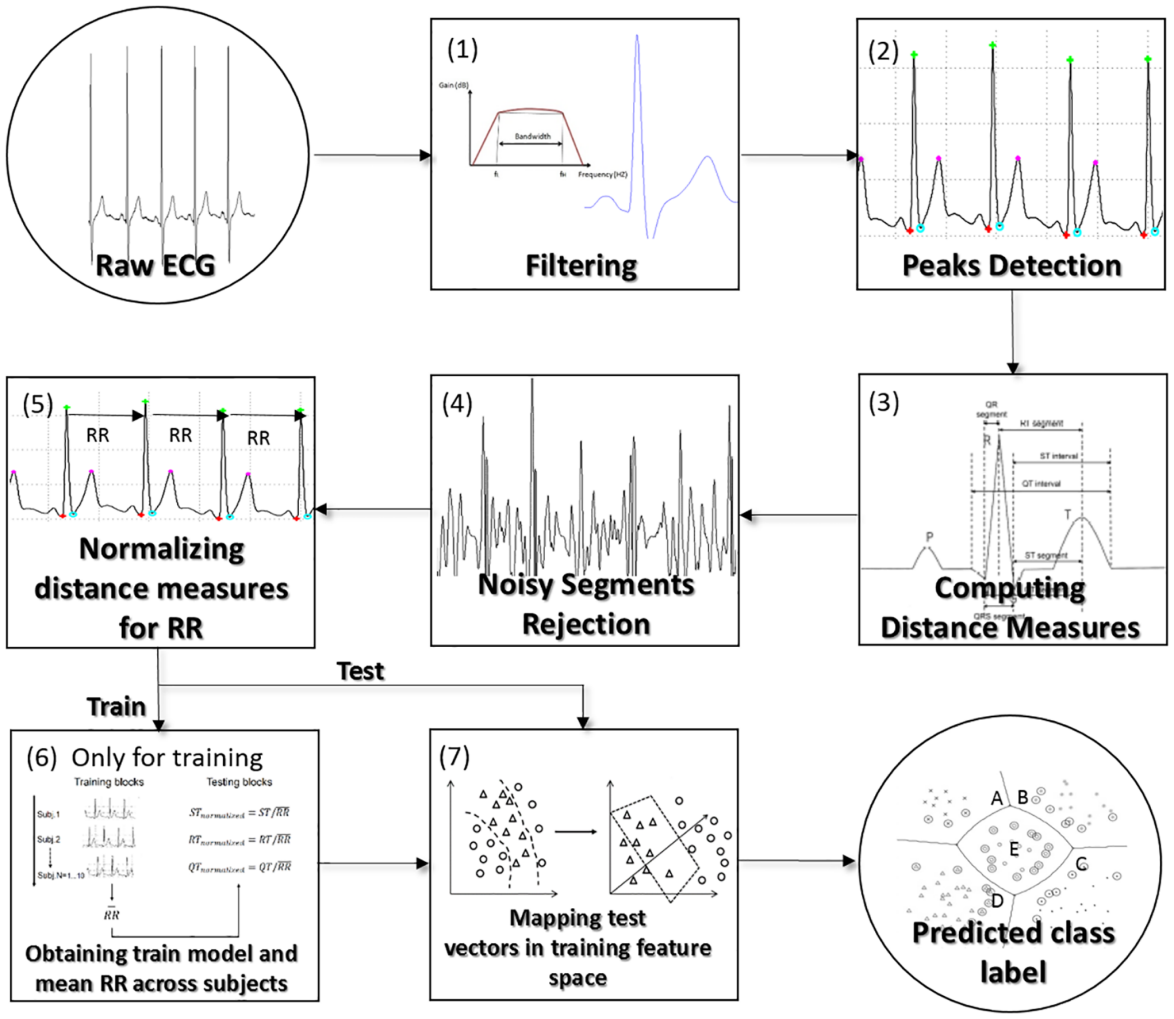
Scheme illustrating the steps of the algorithm. First, the raw signal was filtered (1) and, then, the fiducial points were located (2). After that, the distance measures were computed (3) and noisy heartbeats were removed (4). In the training phase, the distance measures were therefore normalized according to subjects’ heart rate (5). The training features were used to optimize the SVM classifier settings and to build the most suitable training model (6)—part of the figure adapted from [102]. In the test phase, after obtaining the processed data, the test vectors were mapped into the training feature space using the model built during the training phase and the mean RR value across subjects to obtain the predicted label (7).

Our method is characterized by the following steps: ECG preprocessing, feature creation, feature processing, classifier training, and testing. During ECG preprocessing, the fiducial points Q, R, S, and T are detected, after the raw ECG signal is filtered [99]. The R points were located by applying the widely used Pan Tompkins algorithm [100]. The remaining fiducial points (Q, S, and T) were identified after applying a second-order 10-Hz Butterworth low-pass filter to the raw signal and using signal derivatives, as previously performed in other studies [40, 70]. The processing steps involving the location of fiducial points are detailed in subsection **ECG processing: location of ECG fiducial points**. Three features based on the temporal distance between these fiducial points were considered in this *pattern recognition* problem: the ST, RT, and QT distances that characterize each ECG heartbeat, which represents each sample of the dataset considered. After being calculated, the features were processed by way of normalization according to the average distance between each R consecutive points of the training set across all subjects. Heartbeats of which the distance measures provide noisy information (i.e., when the distance measures are not according to previously established physiological limits [68, 101]) were removed, also at the stage at which the features were processed. The most suitable classification model based on the *Support Vector Machine* (SVM) classifier for this specific problem was found at the training stage. In addition, the average RR distance across subjects was used to map future input test vectors into the training features space. The performance of the proposed method was evaluated during the test phase, by considering the information stored during the training phase (best classification model and the training average value of RR across subjects). Additional details about the classification procedure are provided in subsection **Classification Procedure**. Fig 3 presents a scheme summarizing the steps of the algorithm.

### ECG dataset: Physiobank, the Physionet database

The identity recognition method proposed here was validated using the ECG data from 10 subjects available in the Physionet Database (Physiobank) [103]. The Physiobank is a large, growing, and well-characterized database that currently includes more than 60 collections of ECG and other biomedical signals from healthy subjects and patients with a variety of cardiovascular implications [103]. These data are currently considered as the gold standard for the majority of studies based on electrocardiographic signals, and are being used to certify medical software products [103].

The signals from 10 subjects in the PTB Diagnostic ECG Database (*Physionet*)—subjects number 277 (acquisition *s0527*), 234 (acquisition *s0460*), 276 (acquisition *s0526*), 255 (acquisition *s0491*), 247 (acquisition *s0479*), 238 (acquisition *s0466*), 284 (acquisition *s0543*), 233 (acquisition *s0457*), 252 (acquisition *s0487*), and 263 (acquisition *s0499*)—were used here. The PTB Diagnostic ECG Database is a compilation of digitized ECGs for research and algorithmic purposes, collected from healthy volunteers and patients with different heart diseases for the Department of Cardiology of the University Clinic Benjamin Franklin in Berlin, Germany [104, 105]. This database contains records digitized at 1000 samples per second from 290 subjects (healthy and unhealthy) and from 12 leads [104, 105]. The maximal duration of ECG acquisitions contained in the PTB Diagnostic ECG Database is about 2 minutes (120 seconds). Ten healthy subjects whose ECG signals have the maximal duration (two minutes) were randomly chosen for this study. Table 5 presents a description of the dataset that was used.

**Table 5:** Dataset description in terms of gender, mean age of individuals, and length of ECG acquisitions.

Number of Subjects	Gender	Age (y)	ECG length per subject (s)	Total ECG length (s)	Total # analyzed heartbeats
10	9 M/ 1 F	42.88 ± 14.74*	120	1200	1154

*averaged age computed without the information regarding one subject, participant 247, whose age is not included in the *Physionet* database.

Considering the information provided in Table 5, the sample that was used had large age inter-variability, which is an important aspect that can highlight the robustness of the method. Taking into account that significant clinical outcomes are frequently evaluated and detected using ECG signals provided by the lead v5, a derivation widely considered for ECG interpretation schemes [106–108], the ECG signals from the lead v5 were chosen in this study.

Taking into account that the time duration of the ECG acquisitions used here is about 2 minutes (120 seconds), each entire acquisition was randomly split into 12 blocks of 10 seconds per subject, with the respective blocks numbered from 1 to 12. As in a typical *pattern recognition* project, it is necessary to optimize the classification algorithm to find the optimal model for training it. The model is therefore tested using a subset of data independent from those that were used to train the algorithm. Thus, for each person, two non-overlapping blocks of 10 seconds were randomly chosen from the entire acquisition and used to test the classifier. Therefore, for each subject, all the possible combinations between 1 and 12 were chosen for the two blocks ($C_{2}^{12}=66$) to test the classification algorithm. For each one of the 66 combinations, the other 10 blocks that were not selected for testing were used for training the classification algorithm, always ensuring that the order in which data blocks were chosen for training was not repeated between the 66 different combinations, such that the same combination of feature samples was never selected. The algorithm was therefore trained using 10, 20, 30, 40, 50, 60, 70, 80, 90, and 100-second ECG acquisitions, and tested with blocks of 20 seconds (two blocks of 10 seconds each), for 66 runs (each one of the combinations between two testing blocks). We used a 10 seconds increment between the training duration values evaluated because it is a value consistent with the literature around ECG-based recognition methods [36, 75, 98, 109]. Note that the blocks used to test the classifier were never involved in the training, for each one of the 66 combinations. A more detailed description of the training and test sets generation procedure for algorithm evaluation is provided as Supporting Information (section **Supporting information**, **point** S1 Fig).

The following performance measures averaged across the 66 different combinations between the training and test blocks were taken into account in the testing phase of this method: accuracy, False Rejection Rate (FRR), False Acceptance Rate (FAR), and Speed Rate (SR).

### ECG processing: Location of ECG fiducial points

Generally, the recorded ECG signal is often contaminated with noise and artifacts that can interfere with the correct location of fiducial points [3]. Independently of the types of features extracted, an ECG signal with a low signal-to-noise ratio (SNR) could lead to errors in the classifier-training phase and high misclassification rates. Therefore, many different signal processing approaches are reported in the literature [15, 40, 110, 111]. The majority of ECG-based processing schemes include computationally demanding mathematical operations, such as averaging, filtering, wavelet decomposition, among others [15, 38, 112]. In this specific case, the intention was to use as few operations as possible, and also the simplest ones. The signal-processing scheme used in this problem is described in the following paragraphs.

All the signal-processing steps were performed using custom-built MATLAB scripts (MATLAB R2013a, The MathWorks, USA) and specific toolboxes from MATLAB such as the Signal Processing Toolbox and Statistics Toolbox [113].

As mentioned above, the algorithm proposed here only uses the temporal distance between fiducial points. Therefore, the raw signal was processed in order to locate fiducial points with the highest possible precision. The signal processing sequence adopted here is based on former studies that also used features derived from the location of fiducial points [38, 40, 76, 111, 114].

**Fig 4:**
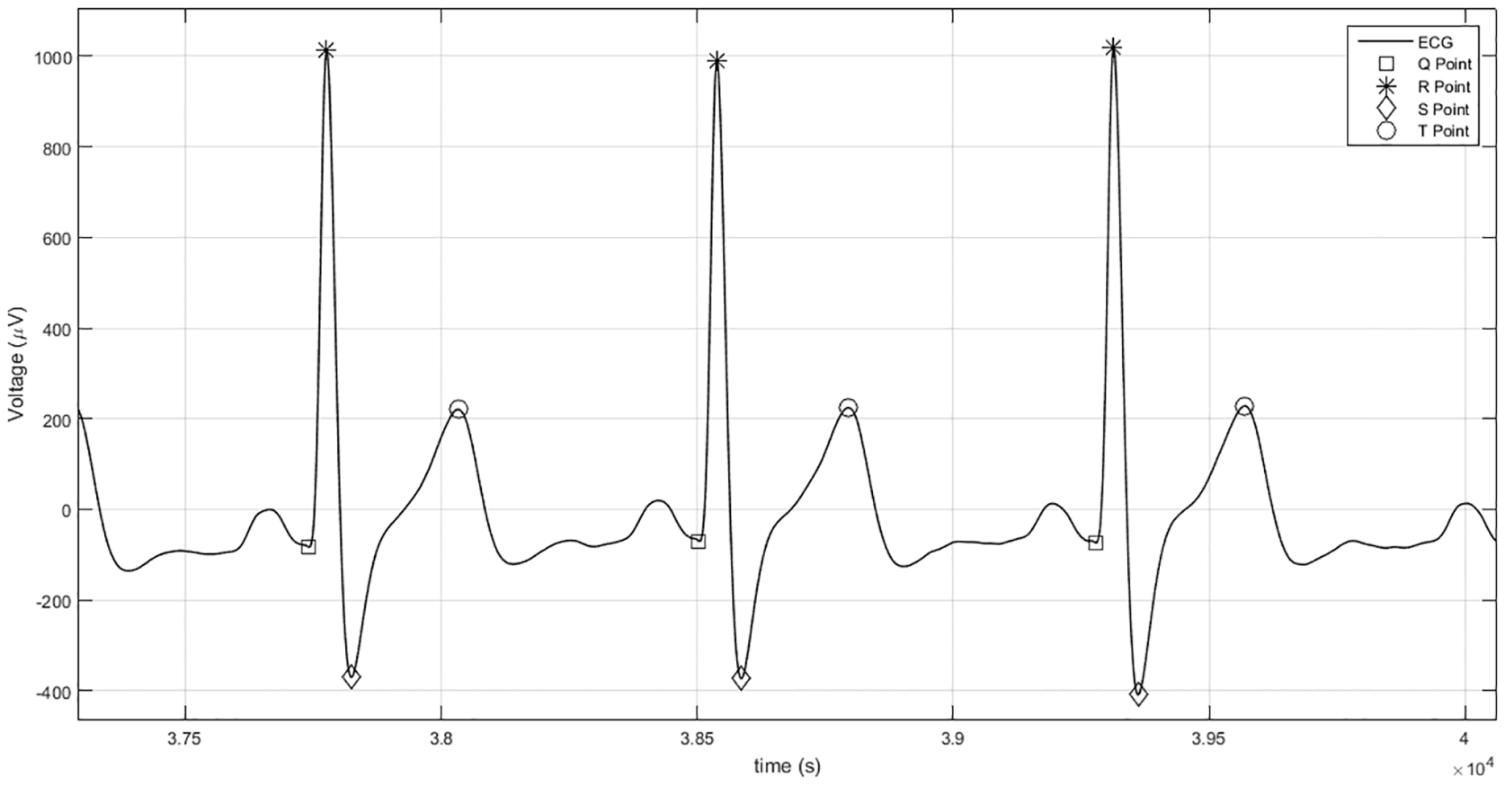
Portion of ECG signal uploaded from the PTB Diagnostic ECG Database processed using the algorithm proposed here. Portion of ECG signal uploaded from the PTB Diagnostic ECG Database processed using the algorithm proposed here—participant number 238 (acquisition reference: *s0466*). Identification of fiducial points Q, R, S, and T.

First, the fiducial points were located on the raw signal to create the features that are used to identify each subject after the raw signal was filtered. Only the complexes Q, R, S, and T were used for feature creation, thus minimizing the computational cost of the algorithm—Fig 4.

The R points were located using the Pan Tompkins algorithm [100], which has been extensively used for the last two decades in the majority of studies related to ECG waveforms [115–118]. It is a real-time algorithm known to reliably recognize R points based upon the digital analysis of the slope, amplitude, and width, relative to the other neighboring fiducials. It consists of several processing steps, starting with the application of a digital bandpass filter that reduces false detections caused by the various types of signals that interfere with ECG signals [100]. According to previous studies, in which the authors examined multiple filtering techniques, the best methods for detecting ECG fiducial points are those based on low-order polynomial filtering [40]. Therefore, the remaining points (Q, S, and T) were identified by filtering the raw signal using a polynomial Butterworth low-pass filter (second order) with a cut-off frequency of 10 Hz—step (1) of Fig 3. The Q points were identified by computing the signal derivative considering a time window of 0.100 seconds defined before each R point. The last temporal mark (and the closest one relative to each R complex) at which the derivative signal crossed zero, considering this time window, was marked as point Q for each heartbeat. Several researchers have been using, in the last decade, derivatives and second derivatives for discovering the location of fiducial points [40, 70]. A similar method was used for locating the point S. The first temporal mark at which the derivative changed from negative to positive values, in a time window of 0.050 seconds defined after each point R, was assigned as the point S. The T wave was located by determining the last temporal index where the derivative of the signal changed from positive to negative values, between 0.050 and 0.400 seconds after each R complex—step (2) of Fig 3. The time windows that were considered to discover each fiducial point were defined based on previously established physiological limits [68]. Therefore, the temporal distances between the referred fiducial points (Q, R, S, and T) were computed for each heartbeat—step (3) of Fig 3, in order to be used as features for the classification task. After calculating all the distance measures, the feature vectors corresponding to noisy heartbeats were rejected by removing the indexes of heartbeats that did not satisfy the following conditions [68, 101]—step (4) of Fig 3:

$$\begin{array}{c} QR\leq 0.075s \end{array}$$ (1)

and:

$$\begin{array}{c} 0.200s<\frac{QT}{\sqrt{RR}}<0.360s \end{array}$$ (2)

The average number of accepted heartbeats across all 66 runs are included in Table 6 (please consult S1 Table in Supporting Information for the mean number of accepted heartbeats for each subject in specific).

**Table 6:** Average number of heartbeats across all 66 combinations.

	Duration (s)	HB Avg. Nr.
**Train**	10	94 ± 1
	20	188 ± 2
	30	283 ± 3
	40	378 ± 4
	50	474 ± 5
	60	569 ± 6
	70	666 ± 6
	80	762 ± 7
	90	859 ± 7
	100	956 ± 7
**Test**	20	181 ± 4

Avg.—average. Nr.—number.

#### Features derived from fiducial points

**Fig 5:**
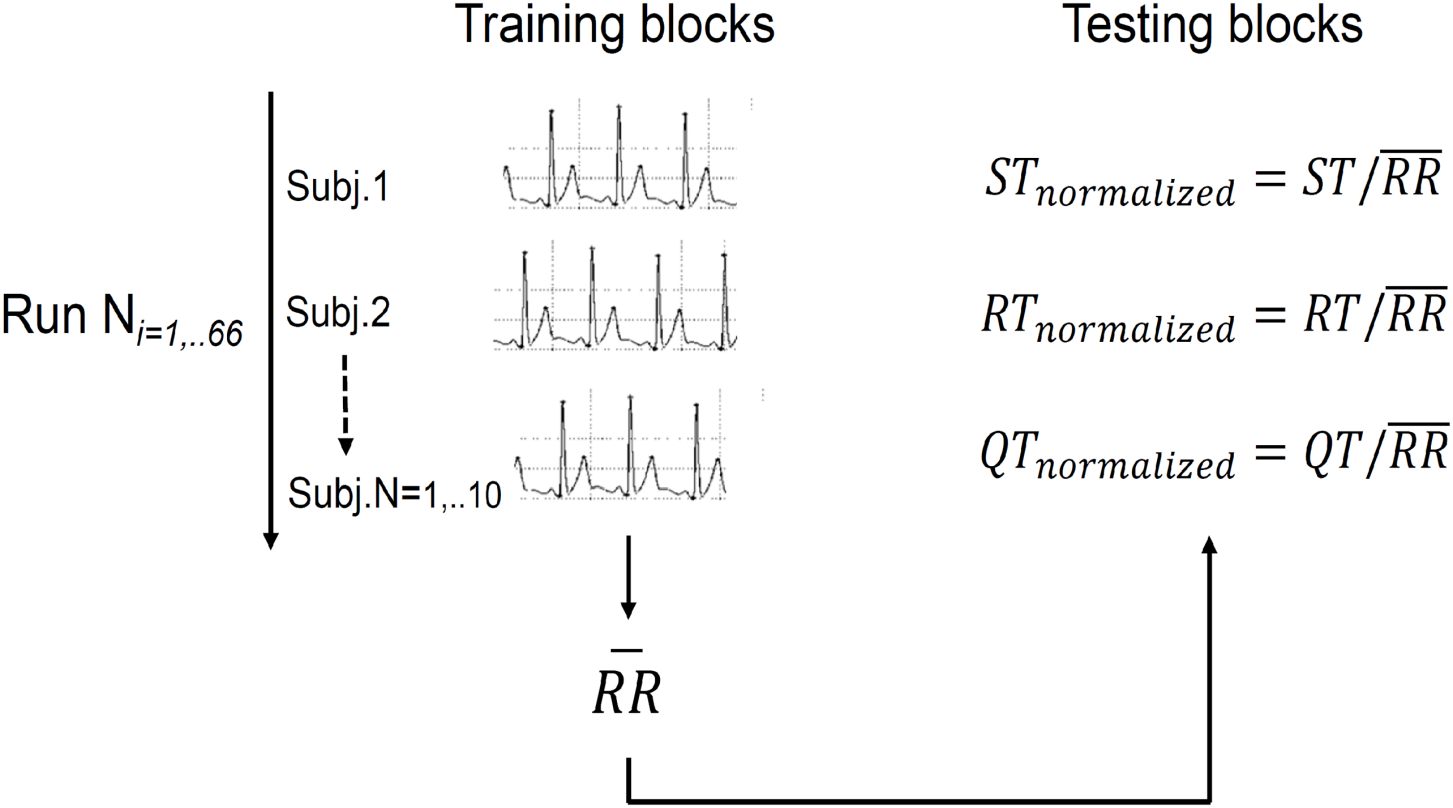
Scheme illustrating the feature normalization procedure, based on the average temporal distance between consecutive R points across subjects. *N* represents the number of combinations (from the 66 between the training and test sets) considered.

The features used in the decision function were based on the temporal distance between the fiducial points Q, R, S, and T—step (3) of Fig 3. Three features were therefore considered: the respective time intervals between Q and T (*QT*), R and T (*RT*), and S and T (*ST*). An independent classification on the subject heart rate required these three features to be normalized (step (5) of Fig 3) using the average RR distance (*RR*) across all subjects in the training set—see Fig 5. Using this normalization method (step (5) of Fig 3), the features provided by a new heartbeat can therefore be normalized and projected into the training feature space independently from the current individual heart rate, on contrary of the majority of the heart rate normalization methods proposed by literature, that use a set of subject-specific RR values for that population [68, 79–82].

### Classification procedure

The classification algorithm used for discriminating each subject through an individual ECG signal was based on a *Support Vector Machine* (SVM), which is a supervised learning algorithm widely used in biomedical applications [119–125]. SVM is currently considered the most adequate type of classifier for biometric applications based on ECG-derived features, due to its ability to cope with the intrinsic nonlinearity of biological data [75, 126]. SVM has also been widely used for developing machine learning-embedded hardware modules, due to its performance, diminished complexity degree in comparison with more complex methods such as Deep Learning algorithms [127], and amount of hardware memory required for the classification task [128, 129]. The literature around this issue showed that SVMs are indeed suitable for hardware incorporation through the years [123, 127–129].

As the SVM is a binary classifier and this specific problem is multiclass, certain procedures must be taken into account in order to convert a multiclass into a binary decision function. The strategy adopted here was to train a single classifier per class, with the samples of that class taken as positive samples and all the other samples as negative ones. Therefore, the final classification rate was assigned to the average between the accuracy values obtained for each one of the binary classifiers developed. This approach is commonly known as *One-Against-All* (OAA) [122, 130, 131].

The performance of the classification task can be maximized by optimizing two general attributes that define the SVM classifier: the hyper-parameter *C*—which controls the trade-off between margin maximization and error minimization—and the *kernel* parameter, which maps the training data into a high-dimensional features space, when the data is nonlinearly separable [119, 121]. There are several types of kernel functions, e.g., the *Radial Basis Function* (RBF), which is the most used and known to generally be more appropriate for biological-derived features, as reported in several previous studies related with ECG and other physiological signals [132–136]. The use of an SVM algorithm with an RBF kernel function also requires a third parameter to be optimized: *σ*, i.e., the width of the Gaussian function. The most accurate classification rate can be obtained by determining the most suitable combination of values between the two hyper-parameters *C* and *σ*. This combination, therefore, produces a classifier trained by considering that pair of values [119, 121]. Different combinations of the parameters C and *σ* were tested here: *C* = {0.01, 0.1, 1, 10, 50, 100, 150, 200} and *σ* = {0.01, 0.1, 1, 10, 50, 100, 150, 200}. The most effective combination of these parameters was determined for each of the 66 different training sets using five-fold cross-validation [119]—step (6) of Fig 3. The input test vectors (for each one of the 66 test sets randomly generated, for each training duration) were normalized by mapping them into the corresponding training features space (step (7) of Fig 3) using the average value of the RR values across subjects obtained in the training stage (see Fig 5). In the training phase, the corresponding mean value of the temporal distance between the R points (*RR*) across all the subjects of each training run was used itself for normalizing each one of the three temporal distance feature vectors. The training samples belonging to a subject were not normalized here by considering only the average value of RR corresponding to that specific subject, because the role of the RR normalization constant was to project a novel ECG heartbeat sample into the training features mapping space of all subjects, independently from the subject to whom this new sample belonged. In fact, the main purpose of this classification method was to identify its user, without that information being given *a priori*—for example, by choosing the corresponding subject-specific RR normalization constant to normalize novel input test samples. This method is therefore advantageous relative to that used by the majority of existing biometric methods regarding its implementation in a low-cost hardware device, which requires a smaller number of normalization constants to be stored on it [135, 137, 138]. This common method is based on features equalization, in which the averaged value across dataset samples along each feature is subtracted from each sample value belonging to that feature and divided by the standard deviation of the feature across samples, implying that it is necessary to store 2*K normalization constants, in which *K* is the number of temporal distance features, in the biometric hardware. In contrast, the normalization method proposed in this paper requires only one normalization constant to be stored in system memory, independently from the number of temporal distance features used.

**Fig 6:**
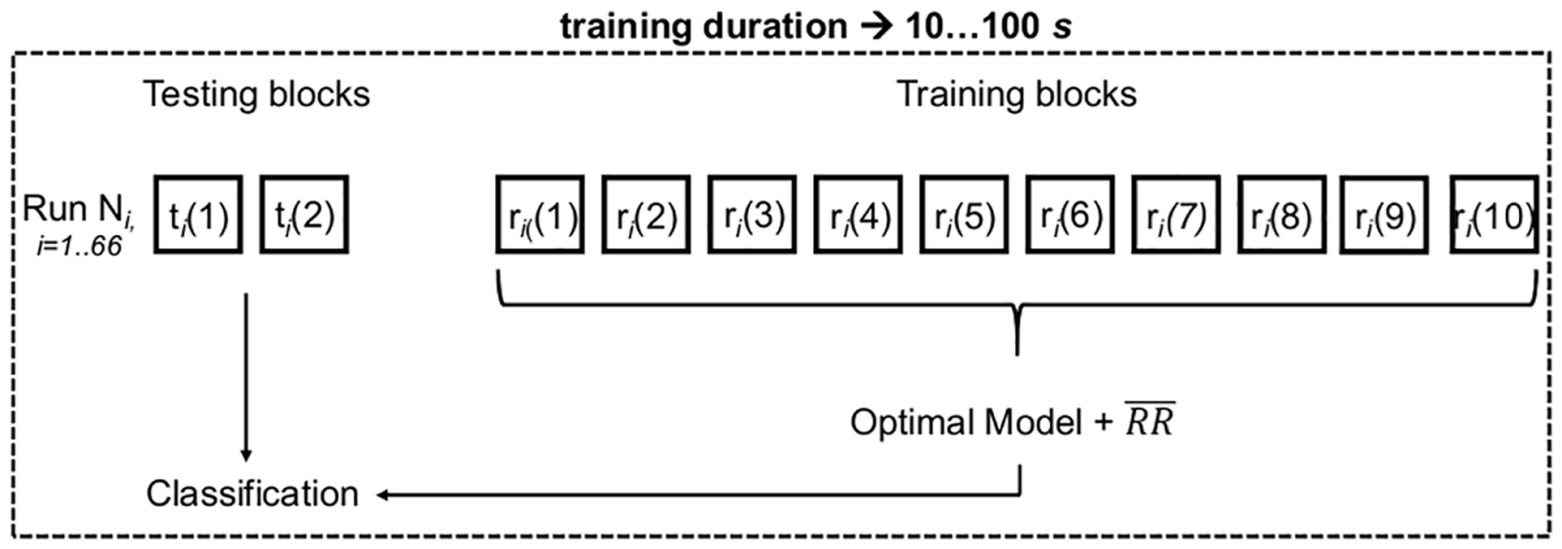
Scheme explaining the classification of heartbeats of each one of the 66 test runs generated for each training duration (10, 20, 30, 40, 50, 60, 70, 80, 90, 100 seconds). *t* represents the data blocks belonging to each one of the 66 test sets; *r* represents each block of the 10 seconds-training set of the 66 sets generated for the duration of each training time duration; *i* represents the number of different combinations evaluated. The procedure illustrated by this scheme was repeated for each training time (10, 20, 30, 40, 50, 60, 70, 80, 90, 100 seconds).

Taking into account that the dataset that was used did not contain a significantly different number of heartbeats (samples) per subject (class)—*p* > 0.05, *n* = 10; two-tailed Kruskal-Wallis—only the total classifier accuracy values were evaluated to determine the classifier performance in the classification task—please see annex S1 Table for a more detailed table including the number of analyzed heartbeats per subject. The input of each one of the 66 test runs was classified by taking into account the training model and information provided from the corresponding training set (one of the 66 runs for each training duration), as explained in Fig 6. A model that was used in one of those 66 runs was never involved in another run.

Apart from analyzing the algorithm performance by taking into account the pre-determined duration for the test sets used in the classification, the minimum number of heartbeats necessary for a positive identification by the algorithm was also evaluated. This required the average number of heartbeats that were used to be determined until the algorithm could correctly identify each subject with 500 repetitions. This procedure was therefore repeated for each one of the 66 combinations that were randomly generated between the test and training sets, for the duration of each training time (10-100 seconds). After each classification run (i.e., by using one of the 66 combinations), the classification algorithm output label was evaluated for each specific subject, taking into account only one heartbeat chosen randomly from the corresponding 20-second test set. If the output label did not correspond to the ground truth, another heartbeat sample that had not been chosen yet was randomly selected from the set of heartbeats, until the classification algorithm correctly identified the current user, or until all the heartbeats in the test set had been used. This procedure was performed 500 times—a limit that is consistent with strategies found in the literature [139, 140]—for each subject and for each one of the 66 combination runs. A description scheme of this algorithm performance evaluation procedure is provided as Supporting Information (S2 Fig).

The performance was evaluated by taking into account the average values of the number of heartbeats necessary to identify each subject across 500 repetitions and along the 66 combinations, for the duration of each training time. The algorithm was evaluated for each one of the 66 combinations between the test and training sets using the parameters *C* and *σ* for which the most accurate cross validation was previously determined.

## Results and discussion

**Fig 7:**
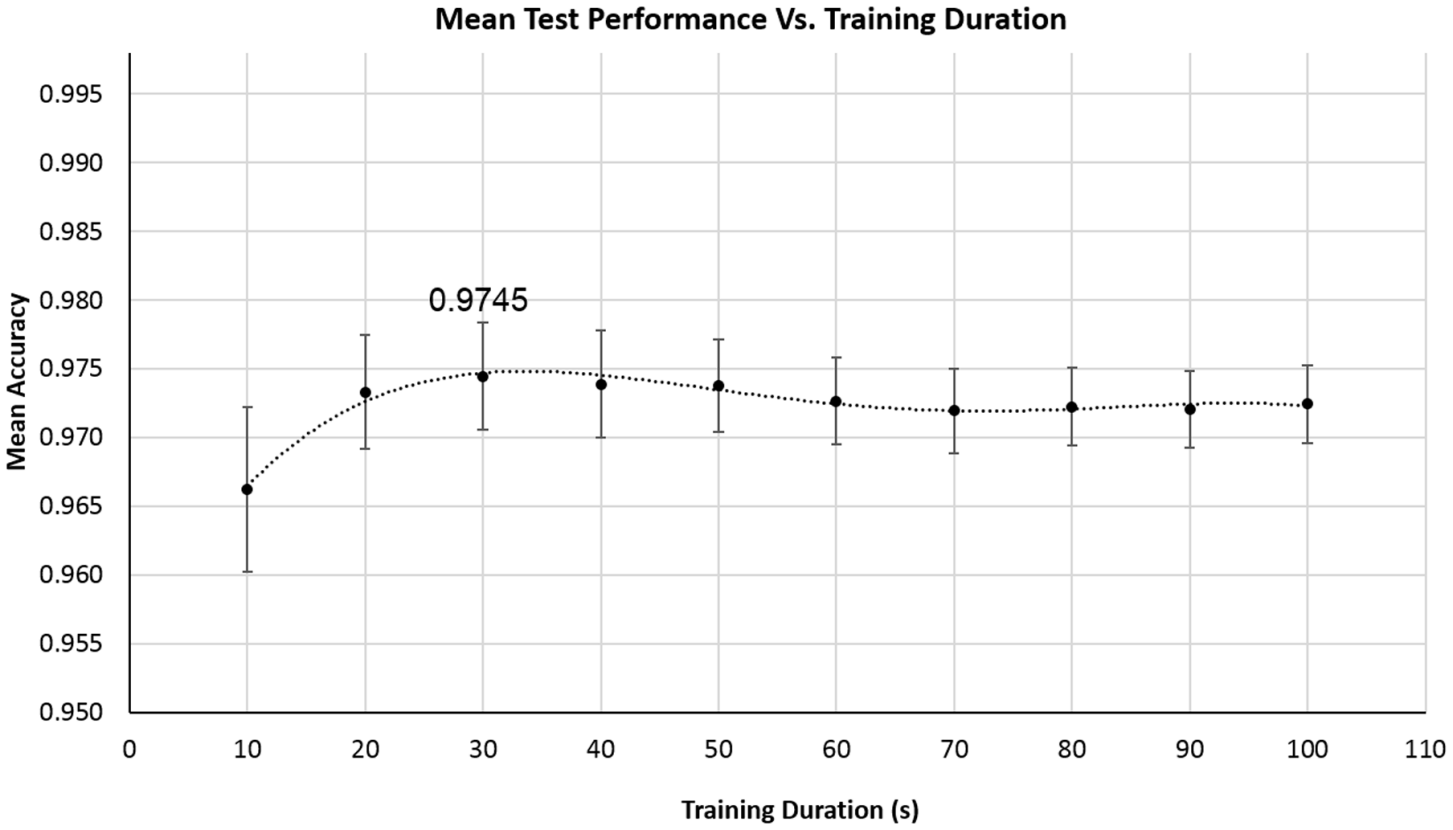
Mean test accuracy obtained for the 66 training and test runs generated, for each training duration, and corresponding standard error bars.

**Fig 8:**
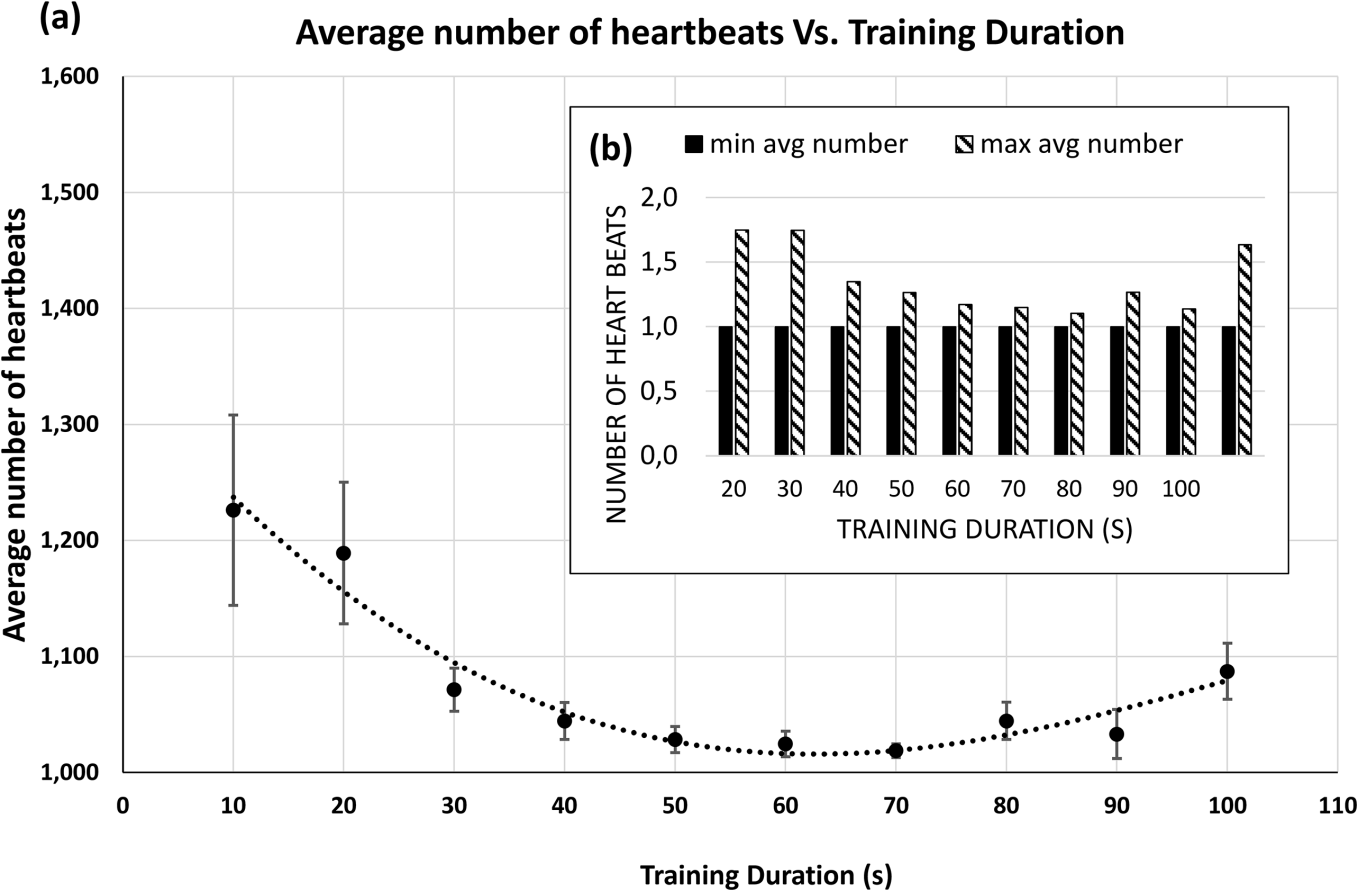
*Beat-to-beat* analysis performance results. a) Number of heartbeats averaged across the 10 subjects and 500 runs necessary to identify each subject individually and the corresponding fitted line; (b) Evolution of maximum and minimum number of heartbeats with the training duration.

**Fig 9:**
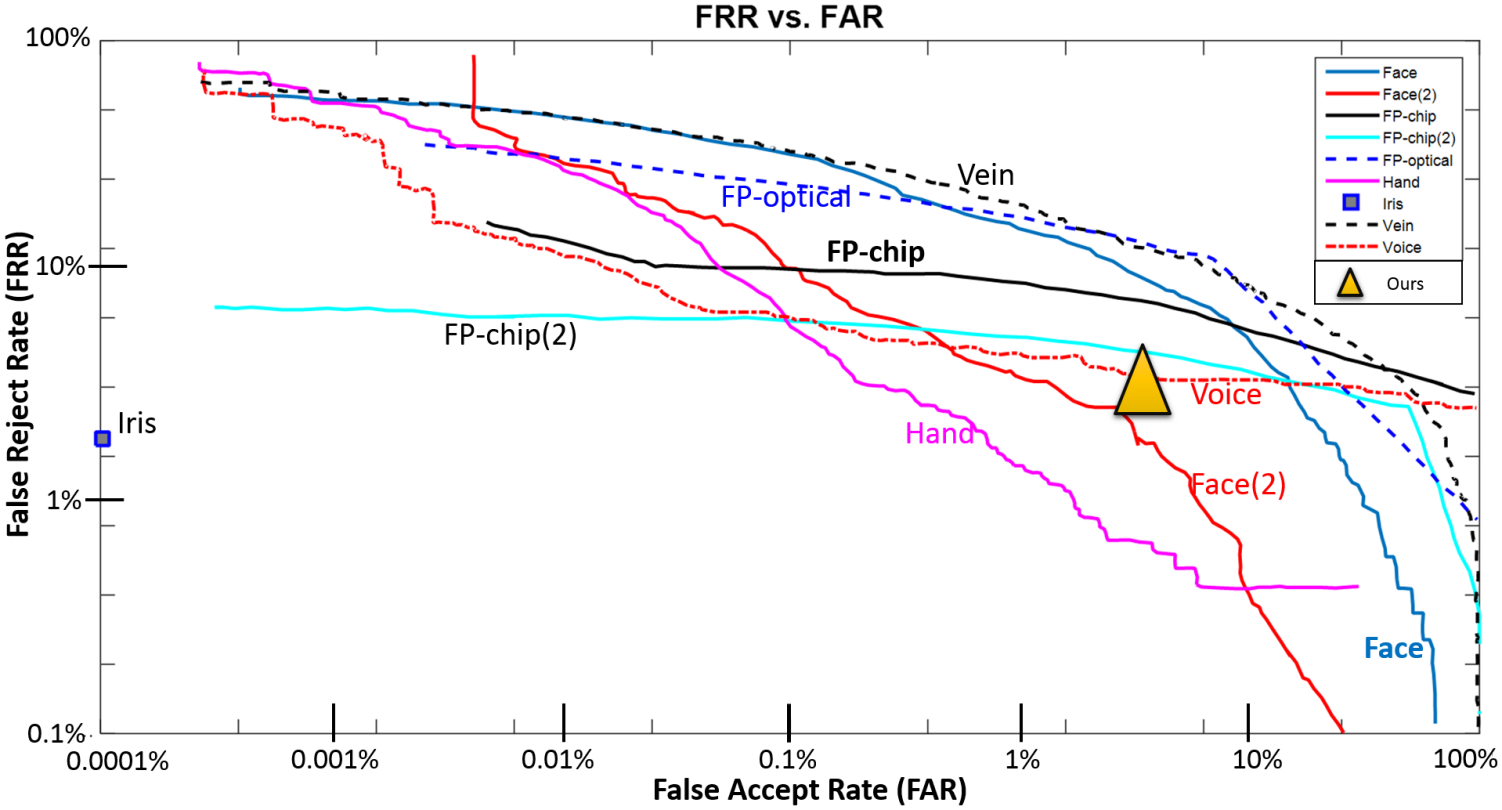
Schematic representation of FRR versus FAR curves for several state-of-the-art techniques in comparison with our method (represented by the yellow triangle). Face and Face(2)—two systems of a face recognition technique validated using different devices; FP-chip and FP-chip(2)—fingerprint recognition through chip sensor tested with two different methods/devices; FP-optical—optical-based fingerprint recognition; Hand—hand-based biometrics; Iris—iris-based recognition; Vein—vein pattern-based recognition; Voice—voice recognition. The devices/systems used to validate each one of these biometric techniques are mentioned and described in the study of Mansfield et al. [17]. Graphic generated considering the results obtained in [17, 141].

Graphical representations of the training results can be found in *Supporting Information*, S3 Fig. Figs 7, 8 and 9 show the test performance results that were obtained. These results reveal that accuracy values higher than 0.960 were obtained by considering different durations for training. The minimal performance value that was achieved was observed for the training set with the shortest duration (the 10-second training set), which ensured an average test performance of 0.966±0.041. It was already expected that the algorithm performance would be less optimal for shorter training sets, since a low number of heartbeats was not considered to be sufficiently discriminative. The average accuracy was observed to improve along with an increase in the duration of the training set. Thus, improved results were obtained by increasing the training time until a plateau was reached, i.e., until the performance values stabilized, and an optimal performance value was reached (a maximal averaged accuracy value of 0.975±0.036), for both of the tested conditions, as depicted in Fig 7. This shows that the optimal testing performance value of 0.975 was achieved for a training duration of at least 30 seconds. Subsequently, this performance value becomes near constant until 100 seconds—the maximal duration of the training evaluation -, suggesting that to train the algorithm for long durations is not worthwhile.

More importantly, the graphic Fig 8 indicates that, globally, the performance achieved by the proposed algorithm is very close to the *beat-to-beat* individual identification for this specific problem. The average number of heartbeats required to correctly identify each person reaches its minimum (1.028±0.092, 1.024±0.089, and 1.019±0.048 for training times of 50, 60, and 70 seconds, respectively, and therefore approximating that of a single beat) at a training time of between 50 and 70 seconds, remaining constant until 90-100 seconds. An inverse relationship between the average number of heartbeats required to correctly identify each person and the duration of the training set is therefore observed, compared to the relation between the classification performance and training duration. According to Fig 8, the average number of heartbeats across the 66 combination blocks that was needed for correct identification among all the subjects was 1.02. This latter analysis determined the minimum number of heartbeats required for the algorithm to achieve a correct identification. This result made it possible to calculate the mean Speed Rate (SR) of the method as being between 0.6 and 1.2 seconds—see Table 7. In general, this SR is shorter, i.e., more optimal, than that of methods developed thus far [17, 18].

**Table 7:** Training estimated optimal parameters for the present method achieving an averaged authentication Speed Rate (SR) of 1.02 heartbeats.

Optimal Parameters		Minimum Authentication Speed Rate (SR)
Features	RBF SVM Training Duration	
1. $ST_{normalized}\;=\;ST/\bar{RR}$	60 seconds	1.02 heartbeats (0.6-1.2 seconds)
2. $RT_{normalized}\;=\;RT/\bar{RR}$		
3. $QT_{normalized}\;=\;QT/\bar{RR}$		

By plotting the averaged FAR and corresponding mean FRR across the 66 different combinations between the training and test sets in the graphic of the Fig 9, it was possible to compare the performance of the proposed method in terms of FAR and FRR with the main existing state-of-the-art techniques. Our method achieved an average value for the FAR and FRR of 5.710±1.900% and 3.440±1.980%, respectively. These results demonstrate that, unlike voice, face, and hand recognition, the proposed method ensures both FAR and FRR within a narrow range of values, demonstrating consistent behavior in performance. Note that our method ensures similar FAR values in comparison with fingerprint verification, the technique currently considered the most mature. However, our method tends to reject fewer input samples and is much less complex and costly.

The normalization method applied here was shown to be innovative, in comparison to state-of-the-art algorithms [68, 79–82], using an average RR value that transduces heart rate-derived information from the subjects’ sample and not several RR values corresponding each to a specific subject, as already discussed before. Despite Gargiulo et al. [75] recently proposing a similar method for correcting variations in ECG due to heart rate variability, our proposed method proved to be more advantageous. They propose several models in order to correct the temporal distance between points Q and T relative to sudden changes in the heart rate, using RR temporal distances. However, their method only included the normalization of one feature—the QT interval—from a set of more than 20 features related to the temporal distance between fiducial points. This is contrary to the algorithm proposed here, which implies a normalization that involves all the fiducial interval features. Additionally, in their method, all the QT interval-related features extracted from the training set that was used were normalized by taking into account the RR distance between two consecutive individual heartbeats of the signal used for testing. Therefore, their algorithm cannot identify a subject using a single heartbeat and in real time, contrary to the method proposed here. The minimum number of heartbeats used to test their biometric method was 200 (approximately 250 seconds, about one hundred times longer than ours).

We conclude that, by combining the results obtained above (Fig 7) with those provided in Fig 8, as summarized in Table 7, near single-heartbeat biometric identity recognition can be achieved by training this algorithm for 60 seconds. Longer datasets are unnecessary to maintain near optimal accuracies. These results are promising, since they were obtained using a homogeneous database composed only of healthy subjects without pathologies that could cause their ECGs to be significantly different from each other.

The performance results achieved by the proposed method are also an important contribution for the understanding of the relevance of electrophysiological intersubject differences, when studying ECG signals provided from different subjects. Indeed, the current study enhanced the importance of the physiological and morphological information which could be derived from the QRS complex and T wave, which, clinically, transduce the ventricular polarization phenomena. Extrapolating for clinical scenarios, if QRST complexes have the information necessary to identify a subject, probably they also have important information for detecting a cardiovascular anomaly, without using other heart cycle-derived measures, which calculus could be more difficult and time consuming (e.g., Heart Rate Variability Measures).

One of the main advantages of the proposed method in comparison with the ones offered by literature is the low complexity of its computational operations, which are based on multiplications, additions, subtractions, and divisions only. This, together with the few features that are used to identify a subject, makes this algorithm suitable for embedding in a simple hardware module for several applications, including entertainment purposes or fast patient recognition by health monitoring systems. Indeed, the low complexity requirements associated with the functioning mode of the proposed method are in accordance with the current limitations of the majority of low-power electronic devices, suggesting that the miniaturization of authentication devices could soon be possible with this approach. As an example, appendix S2 Text contains a description as to how the proposed method was embedded in a simple $3 microcontroller by our research group, leading to the first prototype module of the present system.

In summary, the present study, in addition to provide a less computationally and time consuming biometric method based on a liveness detection technique, is one more proof that ECG morphology analysis could provide valuable information of our cardiovascular system, probably using not more than one or two heartbeats, on contrary of several state-of-the-art methods which includes relatively long RR time series analysis [101].

## Conclusion

A novel and promising method for automatically recognizing a subject using only three characteristics extracted from their ECG waveform that allows fast recognition with high performance rates (around 97.5% of accuracy) and low FRR (of about 3.440±1.980%) was proposed. Owing to its computational simplicity, the proposed method can be embedded in a small device (e.g., a low-cost hardware module), with simple architecture, because it is capable of recognizing a subject by an average of 1.02 heartbeats (requiring only 1-2 heartbeats, approximately 2 seconds maximum), therefore achieving near *beat-to-beat* performance. The method is characterized by two important attributes. The first is the normalization involved in the classification scheme and based on the physiological parameter population-specific RR cardiac length, and the second is the three features based on the ECG morphology that are selected for characterizing each individual.

According to the obtained results, it was concluded that, by training the proposed identity recognition method for 60 seconds, a near single-beat performance recognition algorithm could be achieved, of which the robustness was validated by using 66 different combinations for classification. These results point to the feasibility of developing fast low-power biometric identity check systems using human ECG biosignals.

This study could be also a valuable contribution for enhancing the importance of the morphology-based information that could derived from individuals ECG trace, more concretely, from the QRST complex. This information could be probably helpful for detecting cardiovascular anomalies without the analysis of other difficult and time-consuming heart-derived measures.

To the best of our knowledge, no other study has led to the proposal of such a high-accuracy method capable of identifying a person using fewer heartbeats, or using as few features with such low computational complexity as the method proposed here. In fact, this method presents a Speed Rate (SR) that is comparable to some of biometric state-of-the-art techniques, such for example Hand Geometry Recognition [26–28] or Iris Recognition [21, 29, 30] (please see Table 1), which are much more computationally demanding, and better than more mature methods of literature, such as Fingerprint Recognition [16, 31, 32]. Additionally, it is able to provide liveness detection, on contrary of state-of-the-art techniques (e.g., Facial Recognition [19, 22]; Signature Recognition [20] or Fingerprint Recognition [16, 31, 32]), ensuring similar performance values in comparison with the latter. Lastly, the performance achieved by this method in terms of the FRR/FAR ratio places the Beat-ID in a very competitive position relatively to the majority of the remaining techniques, which present a highly variable FAR *versus* FRR performance behavior (e.g., Fingerprint, Voice or Vein Pattern Recognition). Beat-ID FRR/FAR is, in average, similar but much less variable than Fingerprint Recognition, less computationally costly and less expensive.

Considering that a system able to change its physical and/or virtual configuration by sensing the class of or the specific individual holder through biometric sensing has not been developed yet, our algorithm could greatly advance the development of such a system. It could be embedded in a simple wearable device for applications such as entertainment or gaming, with the ability to interact with its user by changing its shape, rugosity, softness, and button characteristics (color, shape, hard/soft touch) when detecting a specific class (e.g., age group, gender, weight, and height group) or individual holder from a restricted group of persons (for example, a typical family of 6-7 members) through ECG-based information. In future, this method could be embedded in many different “smart objects” for the large IoT market that could benefit from very fast single-beat biometric identity checking (see appendix S2 Text for a simple example) and used for other applications beyond entertainment, such as healthcare and security purposes. This prompted our recent filing of a patent application regarding the proposed method [142].

## Supporting information

S1 Fig Scheme explaining how the training and test sets were obtained. *N* represents the number of combinations between the training and test sets (in this case 66).
(TIF)

S2 Fig *Beat-to-beat* performance evaluation. Scheme illustrating the *beat-to-beat* testing procedure for an example of a test set composed by five heartbeats. Considering each subject, for each one of the 66 combinations between the test and training sets for each training duration: *t* represents the data blocks chosen to be part of the test set; *r* represents each block selected for generating the correspondent training set; *h* represents the set of heartbeats that results from joining the two 10-seconds blocks—*t(1)* and *t(2)*—to generate the test set. Supposing that *h* contains five heartbeats in this example case, *i* represents the position of each heartbeat of *h* in the test set that was generated by joining data blocks *t(1)* and *t(2)*; H_*i*_ represents the i^*th*^ heartbeat of the test set—as, for example H1 is the first heartbeat of the test set -; and *rep* is the pre-determined number of repetitions for which this procedure has to be run for each one of the 66 combinations. The variable *count* is incremented every time the algorithm fails to detect someone using a given heartbeat, until the total number of heartbeats is equal to those in the test set (in the example case illustrated in the figure, this number is 5). If the subject is wrongly identified using a given heartbeat (for example *H1* for the first repetition—*rep = 1*), the algorithm uses the following heartbeat—in this specific case, H2—to identify the subject. If the subject is correctly identified or all the heartbeats were already used separately in an attempt to identify the subject (*count* = 5, in this specific case), the variable *rep* is incremented and the heartbeats belonging to the test set are reordered—to ensure that the sequence of heartbeats chosen to separately identify the subject is not the same as any one of those used in all 500 repetitions.
(TIF)

S3 Fig Algorithm training performance obtained with the five-fold cross-validation method. (a) Mean accuracy training across the 66 training runs generated and corresponding standard error bars. (b) Variation (in terms of standard error values) of the averaged training accuracy across the 66 training runs generated. The training accuracy does not vary considerably for the different training durations. These results show that the training performance was slightly more accurate for shorter training sets, but it can be observed in both graphics (a and b) that training sets with fewer samples and of shorter duration resulted in higher variability of the training accuracy. The standard error in the training accuracy along the 66 different combinations of training data blocks varies according to the duration of the training set, diminishing with an increase in the training duration. In fact, a statistically significant negative correlation between the training duration and standard error of the training accuracy was found along the 66 different combinations of training blocks (*r* = −0.960, *p* < 0.001; *Spearman Test*, two-tailed). The difference between the maximal and minimal value for the mean training performance (across the 66 different runs) along the different durations used for the training set is about 1%. This result together with the finding that the variability in the accuracy training is significantly lower for longer training sets, suggests that it is more advisable to train the model for an intermediate time duration (between 40 and 60 seconds).
(TIF)

S4 Fig Beat-ID component diagram regarding both hardware modules developed. (TIF)

S5 Fig Size evolution of ECG acquisition hardware modules developed, in comparison with a one Euro coin. (TIF)

S6 Fig Workflow diagram illustrating the ECG acquisition and analysis stages embedded in both prototypes. ECG samples are continuously being acquired. When the maximum number of samples is reached (*N* = 350-370), the correspondent ECG segment is immediately analyzed while a new segment is being acquired. The embedded algorithm uses SVM classifier parameters in the subject identification task. These parameters were externally generated in the previous training phase.
(TIF)

S7 Fig ECG waveform captured by the first version of the prototype after being filtered. Location of fiducial points provided by the morphology detection method of the algorithm embedded in the microcontroller at 125 Hz.
(TIF)

S8 Fig Procedure sequence scheme regarding demonstration provided in video S1 Video, including *Realterm* window showing several identification labels sent via Bluetooth from the first version of the prototype. (**12EE**—no identification; **12AA**—subject A identified; **12BB**—subject B identified).
(TIF)

S1 Text Description of training and test dataset generation procedure of the algorithm. (PDF)

S2 Text Beat-ID embedded implementation in a $3 16-bit microcontroller—8KB RAM, 64KB program memory. (PDF)

S1 Table Average number of heartbeats across all 66 combinations between the training and test sets for the duration of each training and testing run and for each class. (PDF)

S1 Video Beat-ID embedded implementation in a $3 16-bit microcontroller—8K RAM, 64K program memory (first prototype developed). The first subject the system attempted to identify is subject B. After a few attempts, the system was able to identify them. Therefore, the second subject, subject A, holds the contact pads for subsequent identification by the system, retrieving from the system a positive identification. *Realterm* was used as a serial terminal application for both acquiring Bluetooth data from the prototype, and for visualizing the subject identification label provided by the system.
(MP4)
