# Supplementary figures and images for: Beat-ID: Towards a computationally low-cost single heartbeat biometric identity check system based on electrocardiogram wave morphology
Source: PLoS One. 2017 Jul 18;12(7):e0180942. doi: 10.1371/journal.pone.0180942 (PMC5515426; doi:10.1371/journal.pone.0180942)

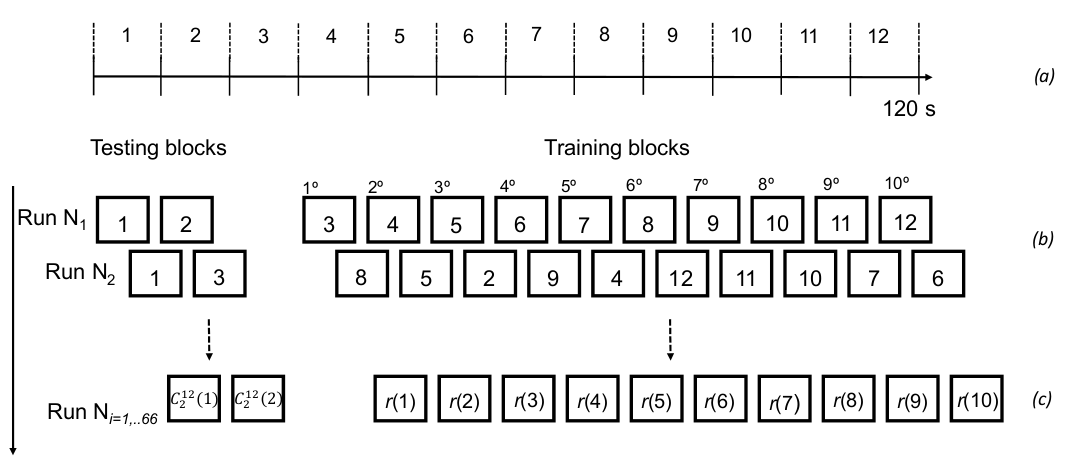

Supplement: S1 Fig — N represents the number of combinations between the training and test sets (in this case 66). (TIF) [file pone.0180942.s001.tif]

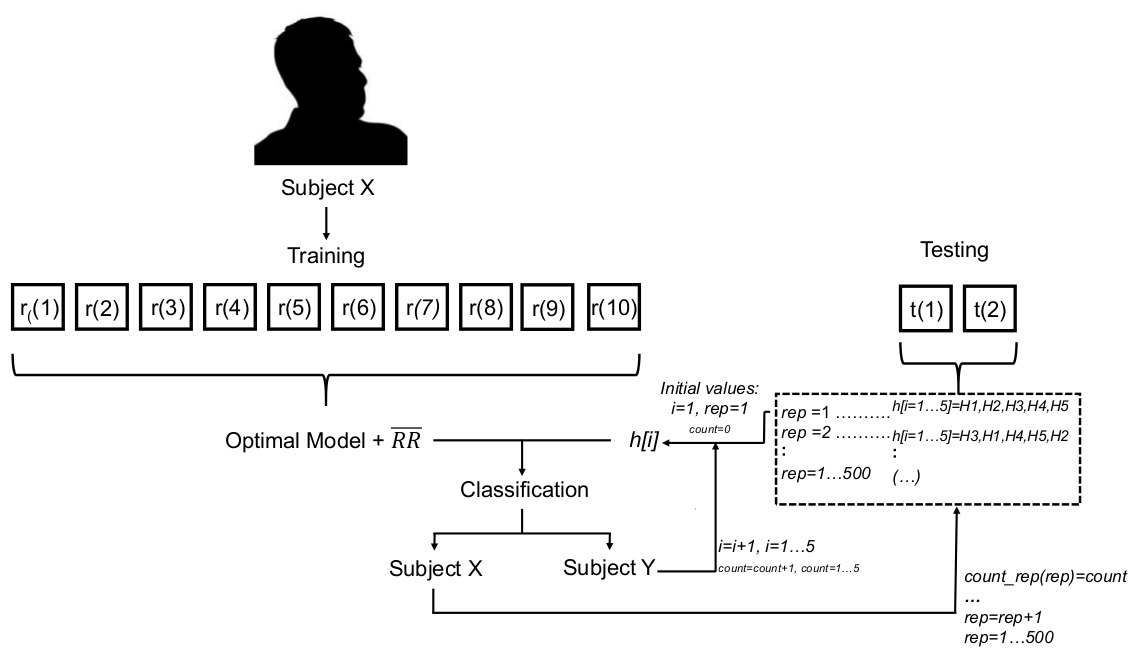

Supplement: S2 Fig — Scheme illustrating the beat-to-beat testing procedure for an example of a test set composed by five heartbeats. Considering each subject, for each one of the 66 combinations between the test and training sets for each training duration: t represents the data blocks chosen to be part of the test set; r represents each block selected for generating the correspondent training set; h represents the set of heartbeats that results from joining the two 10-seconds blocks—t(1) and t(2)—to generate the test set. Supposing that h contains five heartbeats in this example case, i represents the position of each heartbeat of h in the test set that was generated by joining data blocks t(1) and t(2); Hi represents the ith heartbeat of the test set—as, for example H1 is the first heartbeat of the test set -; and rep is the pre-determined number of repetitions for which this procedure has to be run for each one of the 66 combinations. The variable count is incremented every time the algorithm fails to detect someone using a given heartbeat, until the total number of heartbeats is equal to those in the test set (in the example case illustrated in the figure, this number is 5). If the subject is wrongly identified using a given heartbeat (for example H1 for the first repetition—rep = 1), the algorithm uses the following heartbeat—in this specific case, H2—to identify the subject. If the subject is correctly identified or all the heartbeats were already used separately in an attempt to identify the subject (count = 5, in this specific case), the variable rep is incremented and the heartbeats belonging to the test set are reordered—to ensure that the sequence of heartbeats chosen to separately identify the subject is not the same as any one of those used in all 500 repetitions. (TIF) [file pone.0180942.s002.tif]

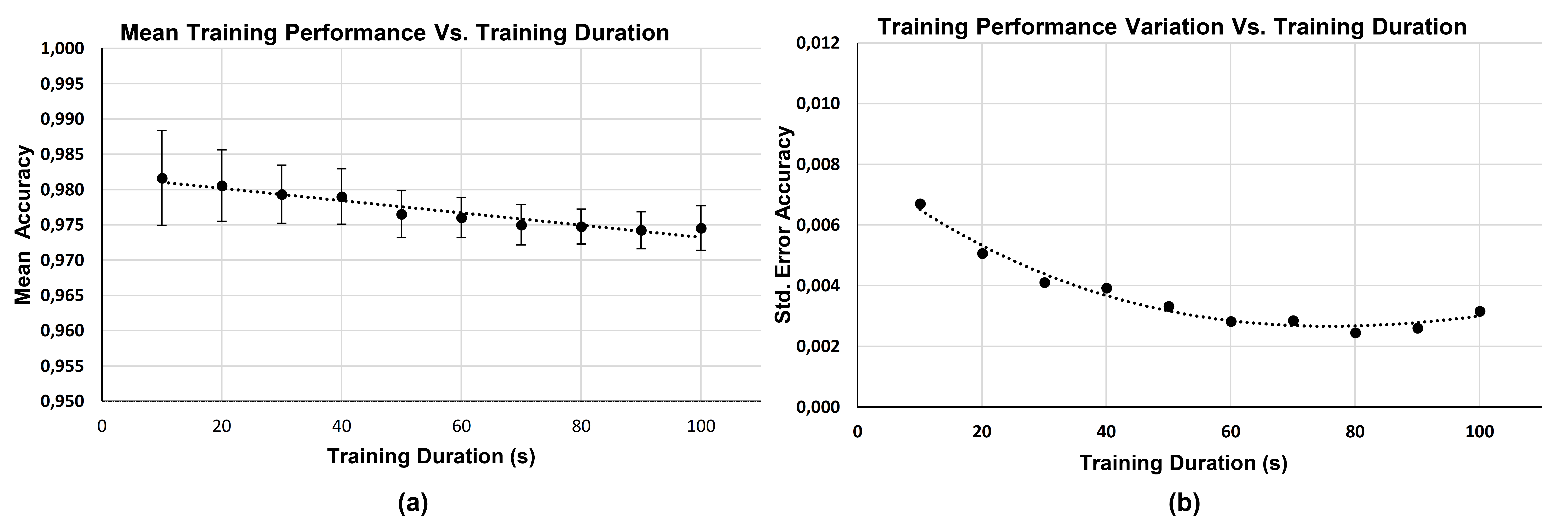

Supplement: S3 Fig — (a) Mean accuracy training across the 66 training runs generated and corresponding standard error bars. (b) Variation (in terms of standard error values) of the averaged training accuracy across the 66 training runs generated. The training accuracy does not vary considerably for the different training durations. These results show that the training performance was slightly more accurate for shorter training sets, but it can be observed in both graphics (a and b) that training sets with fewer samples and of shorter duration resulted in higher variability of the training accuracy. The standard error in the training accuracy along the 66 different combinations of training data blocks varies according to the duration of the training set, diminishing with an increase in the training duration. In fact, a statistically significant negative correlation between the training duration and standard error of the training accuracy was found along the 66 different combinations of training blocks (r = −0.960, p < 0.001; Spearman Test, two-tailed). The difference between the maximal and minimal value for the mean training performance (across the 66 different runs) along the different durations used for the training set is about 1%. This result together with the finding that the variability in the accuracy training is significantly lower for longer training sets, suggests that it is more advisable to train the model for an intermediate time duration (between 40 and 60 seconds). (TIF) [file pone.0180942.s003.tif]

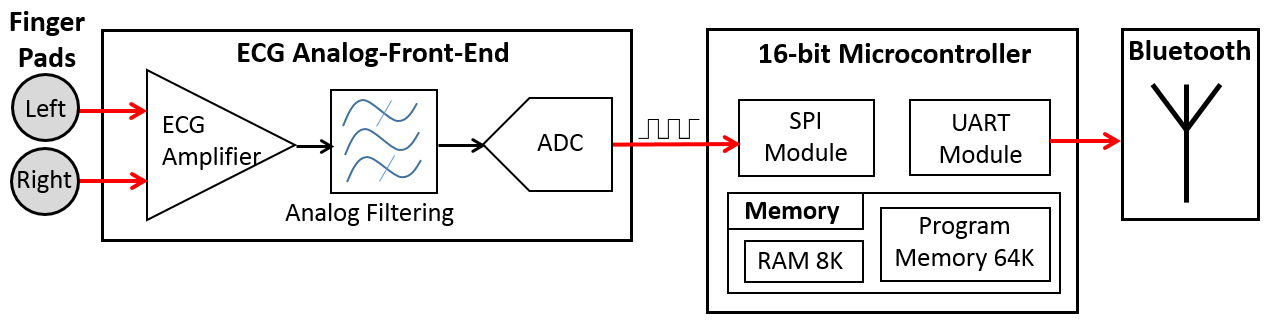

Supplement: S4 Fig — (TIF) [file pone.0180942.s004.tif]

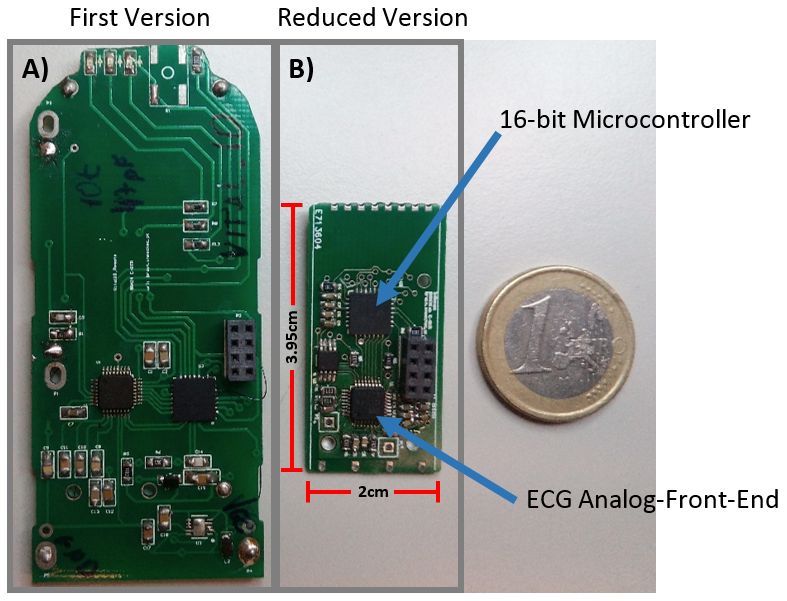

Supplement: S5 Fig — (TIF) [file pone.0180942.s005.tif]

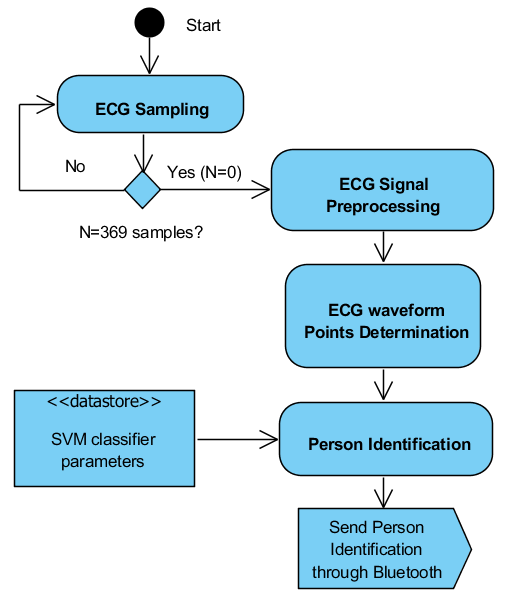

Supplement: S6 Fig — ECG samples are continuously being acquired. When the maximum number of samples is reached (N = 350-370), the correspondent ECG segment is immediately analyzed while a new segment is being acquired. The embedded algorithm uses SVM classifier parameters in the subject identification task. These parameters were externally generated in the previous training phase. (TIF) [file pone.0180942.s006.tif]

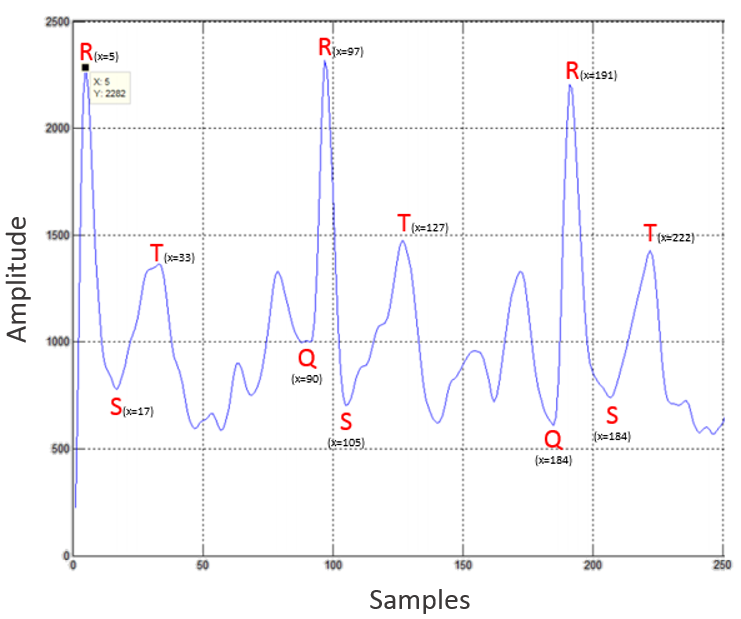

Supplement: S7 Fig — Location of fiducial points provided by the morphology detection method of the algorithm embedded in the microcontroller at 125 Hz. (TIF) [file pone.0180942.s007.tif]

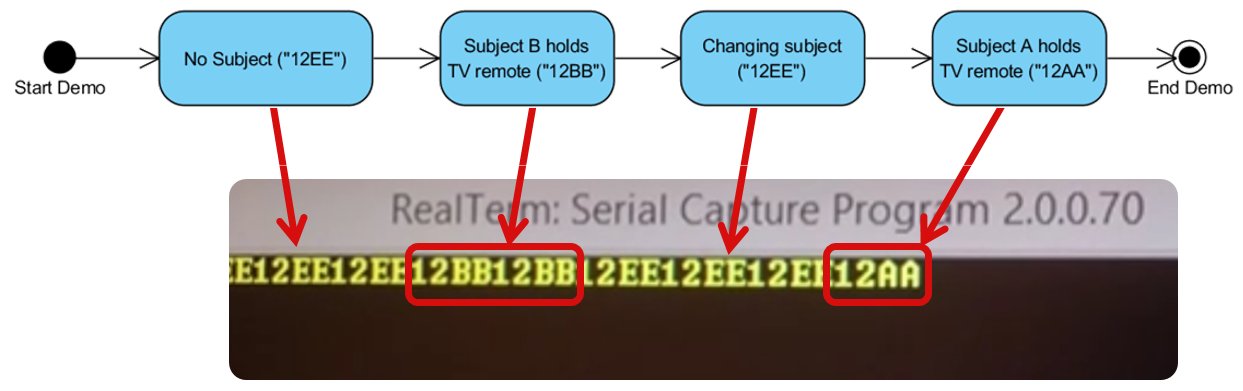

Supplement: S8 Fig — (12EE—no identification; 12AA—subject A identified; 12BB—subject B identified). (TIF) [file pone.0180942.s008.tif]
