# Supplementary material for: Beat-ID: Towards a computationally low-cost single heartbeat biometric identity check system based on electrocardiogram wave morphology
Source: PLoS One. 2017 Jul 18;12(7):e0180942. doi: 10.1371/journal.pone.0180942 (PMC5515426; doi:10.1371/journal.pone.0180942)
Supplement: S1 Text — (PDF) [file pone.0180942.s009.pdf]

S1 Text. Description of training and test dataset generation procedure of the algorithm.
At first, each 120-second acquisition was divided into 12 blocks of 10 seconds each, with each block being numerated as shown in S1 Fig.*(a)*. The testing sets were the first to be chosen and were separated from the other blocks, in order to generate non-overlapping training and testing sets. Therefore, for each subject, all possible combinations between 1 and 12 of 2 blocks ($C_2^{12} = 66$) were chosen for testing the classification algorithm - for example, if the combination of block number one plus block number two was chosen, the feature samples of the first block were combined with the feature samples of the second one for testing, as illustrated in steps S1 Fig.*(b)* and S1 Fig.*(c)*.

For each one of the 66 combinations, the other 10 blocks that were not selected for testing were used to train the classification algorithm. The algorithm was therefore trained using 10, 20, 30, 40, 50, 60, 70, 80, 90, and 100-second ECG acquisitions, and tested considering ECG acquisitions with a time duration of 20 seconds (two blocks of 10 seconds each), for 66 runs (each one of the combinations between two testing blocks). Therefore, for each one of the 66 runs, the training feature samples were generated by joining two, three, or four blocks of 10 seconds for training the algorithm for 20, 30, or 40 seconds and so on, until 10 blocks were reached for training (100 seconds) - the maximal duration for which the algorithm could be trained, considering the use of non-overlapped training and testing sets. The positional order of training blocks was considered for generating the training sets of different duration. Therefore, as an example, for a given combination of two blocks for testing the algorithm, the blocks that would be joined to generate the feature vector relative to 30-second training, were the blocks randomly chosen to occupy the first, second, and third position of the 10-block vector presented in S1 Fig.

Taking into account that it was supposed that, for each one of the 66 runs, a different combination between blocks should be considered for each training set of different duration; thus, if the same block was selected to occupy the same position in two different runs, the correspondent sequence of blocks was randomly reordered for as many times as needed in order to ensure that none of the blocks that were selected occupied the same position as in a former run. The use of this routine guaranteed that the same combination of feature samples would not be selected for training the classifier for several runs.
