# Supplementary material for: Beat-ID: Towards a computationally low-cost single heartbeat biometric identity check system based on electrocardiogram wave morphology
Source: PLoS One. 2017 Jul 18;12(7):e0180942. doi: 10.1371/journal.pone.0180942 (PMC5515426; doi:10.1371/journal.pone.0180942)
Supplement: S2 Text — (PDF) [file pone.0180942.s010.pdf]

S2 Text. Beat-ID Embedded Implementation in a $3 16-bit microcontroller - 8KB RAM, 64KB Program Memory

We demonstrated that the proposed method has the required low complexity to enable it to be embedded in a simple, very low-cost microcontroller and describe in this subsection the main features of two prototypes developed in our laboratory.

The hardware architecture of the prototypes (**S4 Fig.**) was designed to acquire the ECG at the fingers of a subject using an Analog-front-End (AFE) for ECG acquisition, a low-cost microcontroller for running the Beat-ID algorithm and a Bluetooth module to transmit the subject identification data.

We implemented two hardware prototypes in which the proposed method was embedded. The first (**S5 Fig., A**) is a full implementation of the hardware architecture defined above. The second prototype (the smaller one, **S5 Fig., B**) is a miniaturized evolution of the initial one to be inserted into an "intelligent" TV Remote Control that automatically identifies the holder from their touch and automatically adapts its key configuration to its holder. This smaller version does not include the Bluetooth module. Both hardware prototypes were developed around a 16-bit microcontroller with a program memory of 64 KB and 8 KB RAM, that retails for $3 per unit (much lower prices can be achieved for large quantities). To save memory, the ECG sampling frequency was reduced from 500 Hz to 125 Hz and a signal buffer of 370 samples was used, thereby guaranteeing a minimum of two heartbeats to be analyzed during each processing loop. The Beat-ID algorithm implemented in the microcontroller (**S6 Fig.**) and its parameters were adapted to the reduced sampling frequency (e.g., the parameters of the signal filters and the limits of the physiological "windows").

We verified the correct performance of the embedded algorithm by recording examples of the ECG signal and the positions of the fiducial points in the RAM of the microcontroller. An example of this signal is presented in **S7 Fig**. We could conclude that the Q, R, S, and T points were correctly identified and the performance was similar to that achieved before. The ability of the algorithm to detect ECG morphology features endorses the robustness of the proposed method, even when the signal sampling frequency is reduced.

S8 Fig. presents the experimental testing procedure and its results to obtain "on-touch" identification regarding ECG acquisition for the identification of two different subjects (A and B). A set of parameters for the algorithm was trained externally and introduced into program memory, which occupied approximately 5 KB, of this hardware version. As depicted in **S6 Fig.**, after acquiring and processing the data of each subject, this module transmitted the user identification label to a simple terminal. The possibilities for this latter parameter could therefore be: "AA," "BB," or "EE," for the subject A, B, or "no-subject," respectively. A demonstration of this test is also provided in the **S1 Video** video.
