# Supplementary material for: Beat-ID: Towards a computationally low-cost single heartbeat biometric identity check system based on electrocardiogram wave morphology
Source: PLoS One. 2017 Jul 18;12(7):e0180942. doi: 10.1371/journal.pone.0180942 (PMC5515426; doi:10.1371/journal.pone.0180942)
Supplement: S1 Table — (PDF) [file pone.0180942.s011.pdf]

S1 Table. Average number of heartbeats across all 66 combinations between the training and test sets for the duration of each training and testing run and for each class.

	Duration (s)	**HB Avg. Nr.**	Avg. S1	Avg. S2	Avg. S3	Avg. S4	Avg. S5	Avg. S6	Avg. S7	Avg. S8	Avg. S9	Avg. S10
**Train**	10	**94 ± 1**	7 ± 0	7 ± 0	11 ± 0	9 ± 0	10 ± 1	11 ± 1	11 ± 0	8 ± 0	8 ± 0	11 ± 1
	20	**188 ± 2**	13 ± 1	15 ± 1	22 ± 1	18 ± 0	21 ± 1	21 ± 1	22 ± 0	16 ± 0	17 ± 1	22 ± 1
	30	**283 ± 3**	20 ± 1	22 ± 1	34 ± 1	27 ± 0	31 ± 2	32 ± 1	33 ± 0	24 ± 0	25 ± 1	34 ± 1
	40	**378 ± 4**	27 ± 1	29 ± 2	45 ± 2	36 ± 0	42 ± 2	43 ± 1	44 ± 0	32 ± 0	34 ± 2	45 ± 2
	50	**474 ± 5**	34 ± 1	37 ± 2	57 ± 2	45 ± 0	52 ± 2	54 ± 1	55 ± 0	40 ± 0	43 ± 2	57 ± 2
	60	**569 ± 6**	41 ± 1	45 ± 3	68 ± 3	54 ± 0	63 ± 3	65 ± 1	66 ± 0	48 ± 0	51 ± 2	68 ± 2
	70	**666 ± 6**	48 ± 2	52 ± 3	80 ± 3	63 ± 0	73 ± 3	76 ± 1	77 ± 0	56 ± 0	60 ± 2	80 ± 3
	80	**762 ± 7**	55 ± 2	60 ± 3	92 ± 3	72 ± 0	84 ± 3	87 ± 1	88 ± 0	64 ± 0	69 ± 2	92 ± 3
	90	**859 ± 7**	62 ± 2	68 ± 3	104 ± 3	81 ± 0	94 ± 2	97 ± 1	99 ± 0	72 ± 0	77 ± 2	104 ± 3
	100	**956 ± 7**	69 ± 2	76 ± 3	116 ± 3	90 ± 0	105 ± 2	108 ± 1	110 ± 0	80 ± 0	86 ± 2	116 ± 3
**Test**	20	**181 ± 4**	13 ± 1	14 ± 1	22 ± 1	18 ± 0	18 ± 3	21 ± 1	22 ± 1	15 ± 1	16 ± 1	22 ± 1

The number of heartbeats (HB) used per subject (per class) was not significantly different between classes ($p > 0.05$; two-tailed; Kruskal-Wallis test). Avg. - averaged. Nr. - number. S1...S10 - subject number 1 through 10.
